# Dietary Intake in Association with All-Cause Mortality and Colorectal Cancer Mortality among Colorectal Cancer Survivors: A Systematic Review and Meta-Analysis of Prospective Studies

**DOI:** 10.3390/cancers12113391

**Published:** 2020-11-16

**Authors:** Tung Hoang, Hyejin Kim, Jeongseon Kim

**Affiliations:** Department of Cancer Biomedical Science, National Cancer Center Graduate School of Cancer Science and Policy, Goyang 10408, Korea; 75256@ncc.re.kr (T.H.); heajene@gmail.com (H.K.)

**Keywords:** colorectal cancer, mortality, meta-analysis, prediagnostic intake, postdiagnostic intake, colorectal cancer survivors

## Abstract

**Simple Summary:**

Given that an extensive range of dietary factors has not been investigated among colorectal cancer (CRC) survivors to date, we carried out a systematic review and meta-analysis to determine the effects of both prediagnostic and postdiagnostic dietary intake on all-cause mortality and CRC-specific mortality among CRC survivors. In total, 45 studies were included in the final analysis of 35 food items, 8 macronutrients, 27 micronutrients, 2 dietary patterns, and 13 dietary indexes in association with all-cause mortality and CRC-specific mortality. We found that an unhealthy dietary pattern increased the risks of both all-cause mortality and CRC-specific mortality. The role of prediagnostic and postdiagnostic intake such as macronutrients and fatty acids could be different in the risk of all-cause mortality. Overall, comprehensive evidence for the effect of substantial numbers of prediagnostic and postdiagnostic dietary items on mortality outcomes is reported in this study.

**Abstract:**

We carried out a systematic review and meta-analysis to determine the effects of both prediagnostic and postdiagnostic dietary intake on all-cause mortality and CRC-specific mortality among CRC survivors. An extensive search of PubMed and Embase was conducted to identify eligible studies. We applied a random-effects model to estimate the pooled relative risks (RRs)/hazard ratios (HRs) and their 95% confidence intervals (CIs). As a result, a total of 45 studies were included in the final analysis. Pooled effect sizes from at least three study populations showed that whole grains and calcium were inversely associated with all-cause mortality, with RRs/HRs (95% CIs) of 0.83 (0.69–0.99) and 0.84 (0.73–0.97), respectively. In contrast, a positive association between an unhealthy dietary pattern and both all-cause mortality (RR/HR = 1.47, 95% CI = 1.05–2.05) and CRC-specific mortality (RR/HR = 1.52, 95% CI = 1.13–2.06) was observed among CRC survivors. In the subgroup analysis by CRC diagnosis, prediagnostic and postdiagnostic dietary intake such as carbohydrates, proteins, lipids, and fiber were observed to have different effects on all-cause mortality. Overall, an unhealthy dietary pattern increased the risks of both all-cause mortality and CRC-specific mortality. The role of prediagnostic and postdiagnostic intake of dietary elements such as macronutrients and fatty acids could be different in the risk of all-cause mortality.

## 1. Introduction

According to the GLOBOCAN study, the estimates of the global burden of cancer were 18.1 million new cases and 9.6 million deaths in 2018 [1]. Among 36 cancer types, colorectal cancer (CRC) is the third leading cause of cancer and the second main cause of cancer-related deaths, with 1.8 and 0.8 million people newly confirmed, respectively, in 185 countries [1], despite a decrease in the years of life lost of 4.7% since 1990 according to the Global Burden of Disease Study in 2017 [2]. Nevertheless, the 1-year and 5-year survival rates of CRC patients have consistently increased for a decade with improvements in the early diagnosis and treatment of the disease and in screening modalities [3]. In particular, 8% of all cancer survivors are CRC survivors (defined as patients who have ever been diagnosed with CRC) [3]. The combination of a healthy diet and lifestyle changes could be a determining factor in decreasing the high burden among CRC survivors, such as by lowering the risk of recurrence or mortality and improving the quality of life after diagnosis, surgery, or chemotherapy. Changing in dietary habits, such as consuming less red and processed meat, dramatically have been shown to occur among CRC patients within the preceding 2 years after CRC diagnosis [4].

According to the most recent report from the World Cancer Research Fund and the American Institute of Cancer Research (WCRF/AICR), red and processed meat and alcoholic beverages have been confirmed to be strong modifiers that increase the risk of CRC [5]. However, whole grains, foods including dietary fiber, and dairy products were linked to a decrease in the risk of CRC, while the effects of other food or nutrient components were undetermined [5]. However, the accumulative evidence from several meta-analyses, and the report from the WCRF/AICR regarding diet and CRC are insufficient to form a conclusion to help CRC survivors cope with any side effects or the threat of cancer recurrence. A few previous meta-analyses of diet and cancer risk among CRC survivors found a decreased risk of CRC-specific mortality with a Mediterranean dietary pattern [6] and an increased risk of CRC-specific mortality with a Western dietary pattern [7]. The limited lifestyle interventions for CRC survivors have focused on multiple health behavioral changes, mainly including diet and physical activity [8]. However, given that an extensive range of other dietary factors has not been investigated among CRC survivors to date, the time when a CRC survivor consumes food and nutrients from various dietary sources (during and/or after diagnosis, surgery, chemotherapy, recurrence, or adverse effects of CRC) could be an important indicator for subsequently improving the patient’s quality of life later on, emphasizing the importance of this meta-analysis of dietary intake, overall mortality, and CRC-specific mortality among CRC survivors. Therefore, this study aimed to perform a systematic review and meta-analysis of prospective studies to gain more comprehensive knowledge regarding the association between dietary intake and all-cause or CRC-specific mortality and to identify the associations that differed per prediagnostic and postdiagnostic dietary intake among CRC survivors.

## 2. Materials and Methods

### 2.1. Literature Search and Study Selection

The review protocol of this study was not registered with PROSPERO. Few studies investigating the association between a single diet (and/or nutrients), dietary patterns or dietary indexes, and CRC risk were published prior to 2000 [9], especially among CRC survivors. Nearly 20 years of evidence-based research might give CRC patients more opportunities to promote the prevention of chronic diseases, including CRC. We therefore searched for and identified eligible studies published from 1 January 2000 to 31 March 2020, via the PubMed and Embase databases by using the following keywords: “(diet or nutrition or dietary or consumption or intake) and (colorectal or colon or rectum or rectal) and cancer and (survival or mortality)”. Details of the search strategy are presented in Table S1. An additional search was conducted on the basis of a review of the references of the retrieved articles and the updated publications between 01 April 2020, and 14 May 2020, after the removal of duplicates.

The inclusion and exclusion criteria for the articles are summarized in Table S2. Briefly, the inclusion criteria for the articles were as follows: (1) the design was a prospective study (cohort study or randomized controlled trial); (2) the exposure of interest was any type of dietary intake (single food items, nutrients, dietary patterns, or dietary indexes); (3) the outcome of interest was mortality due to any cause, CRC, colon cancer, or rectal cancer; (4) a full-text version of the article was available; and (5) the relative risk (RR) or hazard ratio (HR) and their 95% confidence intervals (CIs) were provided. Concerning two similar results from the same cohort study, the study with the larger population or longer follow-up duration was included in the final meta-analysis. The exclusion criteria for the screened articles were as follows: (1) different exposures and/or outcomes; (2) unrelated study design (in vitro, animal, or experimental designs); (3) studies without original data (abstracts or reviews); (4) studies with uncertain information regarding the RR or HR; and (5) overlapping studies. Two reviewers (T.H. and H.K.) independently conducted the literature search and study selection. Any discrepancies were resolved by consultation with a third reviewer (J.K.).

### 2.2. Data Extraction

Two investigators (T.H. and H.K.) independently extracted the data according to the Preferred Reporting Items for Systematic Reviews and Meta-Analyses (PRISMA) statement [10]. Any differences in decision making were addressed, and a consensus was reached through discussion. The data extracted from the full-text articles included the last name of the first author, year of publication, project name, the time point of dietary intake (before or after CRC diagnosis), age at baseline (standard deviation; SD), sample size, sex, exposure (single nutrient or food intake, dietary pattern, or dietary index), outcome (all-cause, CRC, colon cancer, or rectal cancer mortality), RR/HR with the corresponding 95% CI for each category of dietary exposure, and covariates adjusted in the final statistical model in each study.

### 2.3. Quality Assessment

The quality of the studies included in the final analysis was evaluated by using the Newcastle–Ottawa Scale (NOS) for cohort studies [11]. The assessment tool includes a total of 9 items for the selection of study groups, comparability of groups, and ascertainment of the outcome of interest, with a maximum of 9 scores [11]. Studies with a score of greater than 6 were included in the final analysis. As we included prospective studies, we further assessed the attrition bias in all individual studies.

### 2.4. Statistical Analysis

A systematic review of the effect sizes (RR/HR and 95% CI) of any single food items, macronutrients, micronutrients, dietary patterns, and dietary indexes in the association with all-cause mortality and/or CRC-specific mortality was conducted. When at least two studies were available for the measurement of the association between exposures and outcomes of interest, a meta-analysis using a random-effects model was performed by combining the multivariable-adjusted RR/HR of the highest compared with the lowest quartile [12]. Additionally, we examined publication bias by using Begg’s funnel plot and Egger’s test when 4 or more studies were available [13,14]. Subgroup analyses of prediagnostic and postdiagnostic dietary intake were conducted to examine the behavioral differences among CRC patients. The heterogeneity in between-study variation was assessed by calculating the I^2^ statistic, and an I^2^ value greater than 50% suggested substantial heterogeneity [15]. STATA SE version 14.0 (StataCorp, College Station, TW, USA) was used for all statistical analyses.

## 3. Results

### 3.1. Study Selection

**Figure 1:**
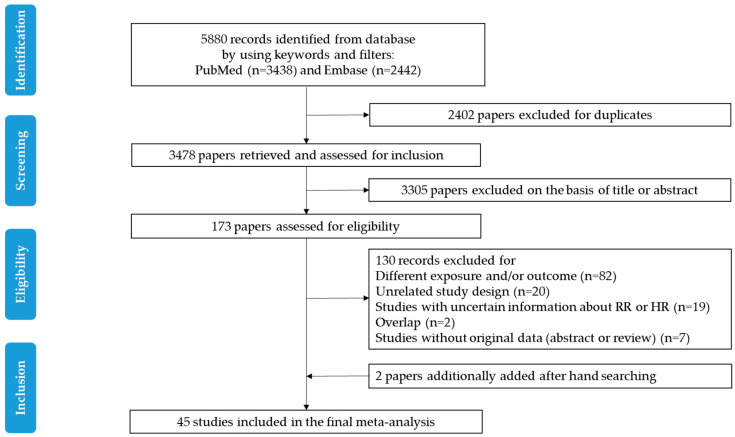
Flow chart of the study selection. The flow chart shows the process used to select prospective studies for the systematic review and meta-analysis of the association between dietary intake and all-cause mortality and colorectal cancer (CRC) mortality among CRC survivors. RR, relative risk; HR, hazard ratio.

The detailed steps of the systematic review and meta-analysis are shown according to the PRISMA flow diagram (Figure 1). Of the 5880 records identified in PubMed and Embase, 3478 papers were assessed for titles and abstracts after the removal of duplicate records. Of these studies, 3305 irrelevant articles were removed; hence, 173 full-text reports were retrieved. Two additional articles were identified by hand searching. Among a total of 175 full-text reports, 130 papers were additionally excluded due to an different exposure and/or outcome (*n* = 82), unrelated study design (*n* = 20), studies with uncertain information regarding RR or HR (*n* = 19), overlap (*n* = 2), or studies without original data (abstracts or reviews) (*n* = 7). As a result, 45 studies were included in the final systematic review and meta-analysis [16,17,18,19,20,21,22,23,24,25,26,27,28,29,30,31,32,33,34,35,36,37,38,39,40,41,42,43,44,45,46,47,48,49,50,51,52,53,54,55,56,57,58,59,60].

### 3.2. Study Characteristics and Quality Assessment

The characteristics of the included studies are summarized in Table S3. All studies were published between 2003 and 2019. The mean/median age of the study population at baseline ranged between 55 and 73 years. Exposure data related to prediagnostic intake only were available in 21 studies [17,18,19,20,28,29,34,35,36,39,40,41,42,43,46,49,50,55,56,57,59], exposure data related to postdiagnostic intake only were available in 20 studies [16,21,22,23,24,25,26,27,31,32,33,37,38,44,45,47,48,51,54,58,60], and 4 studies included both prediagnostic and postdiagnostic dietary intake [26,30,52,53]. Outcome data related to all-cause mortality only were available in 16 studies [16,20,21,22,24,25,31,32,33,36,37,38,41,43,47,48], 8 studies included outcome data related to CRC-specific mortality only [28,29,42,46,56,57,58,59], and 21 studies included both all-cause mortality and CRC-specific mortality [17,18,19,23,26,27,30,34,35,39,40,44,45,49,50,51,52,53,54,55,60].

Table S4 shows the quality assessment of the individual studies according to the NOS. All studies achieved a score of at least 7 out of 9 and were deemed to be high quality. As dietary information was obtained via a food frequency questionnaire (FFQ) without any medical link record, none of the studies achieved a score for the ascertainment of the exposure criteria. Additionally, attrition bias is presented in Table S5.

### 3.3. Main Analysis

Table 1 displays the pooled estimates of the overall (prediagnostic and/or postdiagnostic) food items (*n* = 35), macronutrients (*n* = 8), micronutrients (*n* = 27), dietary patterns (*n* = 2), and dietary indexes (*n* = 13) in the association with all-cause and CRC-specific mortality among CRC survivors. The highest consumption of fruits, whole grains, dark fish, coffee, and calcium was found to be negatively associated with the risk of all-cause mortality by using a random-effects model, with RRs/HRs (95% CIs) of 0.88 (0.80–0.96), 0.83 (0.69–0.99), 0.68 (0.48–0.96), 0.69 (0.55–0.98), and 0.84 (0.73–0.97), respectively. On the other hand, refined grain intake and an unhealthy pattern (high-sugar, processed meat, or Western pattern) were significantly associated with an increased risk of all-cause mortality (RR/HR = 1.88, 95% CI = 1.25–2.85 and RR/HR = 1.41, 95% CI = 1.15–1.73, respectively), but no significant association between a prudent pattern and all-cause mortality was observed among CRC survivors (RR/HR = 0.93, 95% CI = 0.82–1.04). Furthermore, the CRC survivors in the highest category of the American Cancer Society recommendations (ACS score) and the WCRF/AICR score had a 30% (RR/HR = 0.70, 95% CI = 0.56–0.86) and 21% (RR/HR = 0.79, 95% CI = 0.65–0.98) lower risk of all-cause mortality compared with those in the lowest category, whereas the subjects in the highest category of glycemic load and insulin index had a 74% (RR/HR = 1.74, 95% CI = 1.20–2.51) and 51% (RR/HR = 1.51, 95% CI = 1.07–2.12) higher risk of all-cause mortality, respectively.

Regarding CRC-specific mortality, the intake of grilled food and carbohydrates was associated with 78% and 91% increased risks of CRC-specific mortality, with RRs/HRs (95% CIs) of 1.78 (1.05–3.02) and 1.91 (1.17–3.12), respectively. Additionally, the positive association with an unhealthy dietary pattern (RR/HR = 1.52, 95% CI = 1.13–2.06) and negative associations with coffee consumption (RR/HR = 0.47, 95% CI = 0.31–0.71), the ACS score (RR/HR = 0.35, 95% CI = 0.17–0.73), and the WCRF/AICR score (RR/HR = 0.70, 95% CI = 0.56–0.89) remained significant.

**Figure 2:**
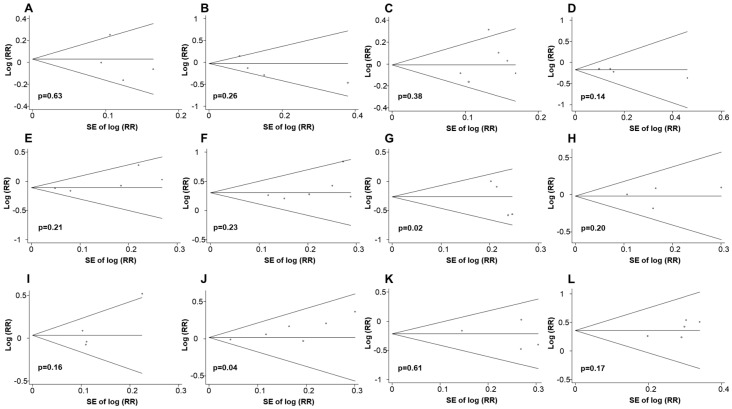
Funnel plot for the analysis of publication bias. (**A**) Red and processed meat, (**B**) total dairy, (**C**) alcohol, (**D**) calcium, (**E**) prudent pattern, (**F**) unhealthy pattern in association with all-cause mortality, (**G**) whole grain, (**H**) red and processed meat, (**I**) red meat, (**J**) alcohol, (**K**) prudent, (**L**) unhealthy in association with colorectal cancer death. RR, relative risk; SE, standard error.

Possible publication bias when four or more studies were available was assessed by Begg’s funnel plot and Egger’s test (Figure 2). Publication bias was observed for the association of dietary whole grain (*p* = 0.02) or alcohol (*p* = 0.04) and CRC death.

### 3.4. Subgroup Analysis

The subgroup analysis of the associations between prediagnostic dietary intake (27 food items, 8 macronutrients, 27 micronutrients, 2 dietary patterns, and 13 dietary indexes) and mortality among CRC survivors is presented in Table 2. The observed pattern of a negative association between fruits and all-cause mortality and of a positive association between grilled foods and CRC-specific mortality was similar to that in the results of the main analysis.

The subgroup analysis for the associations between postdiagnostic dietary intake (15 food items, 8 macronutrients, 2 micronutrients, 2 dietary patterns, and 9 dietary indexes) and mortality among CRC survivors is presented in Table 3. The observed pattern in the association between refined grains, whole grains, dark fish, coffee consumption, ACS score, or glycemic load and all-cause mortality was similar to that of the results from the main analysis.

**Figure 3:**
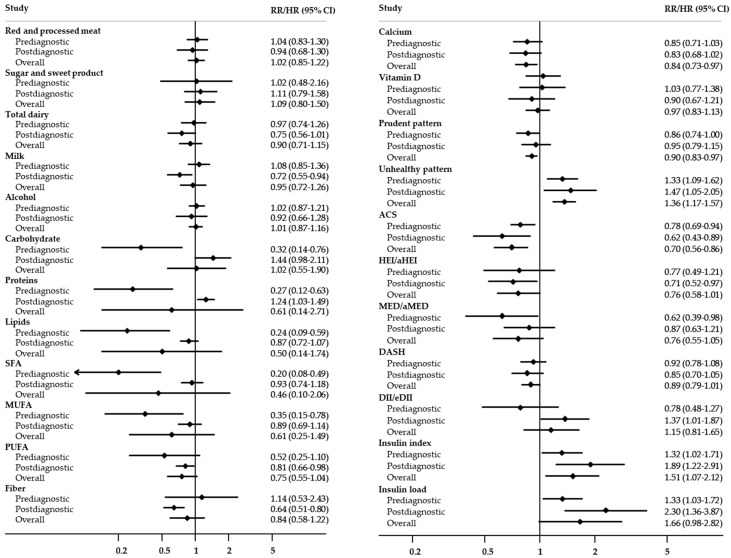
Comparison for the roles of prediagnostic and postdiagnostic intake in all-cause mortality. SFA, saturated fatty acids; MUFA, monounsaturated fatty acids; PUFA, polyunsaturated fatty acids; ACS, American Cancer Society recommendations score; HEI, Healthy Eating Index; aHEI, alternate Healthy Eating Index; DASH, Dietary Approaches to Stop Hypertension score; DII, Dietary Inflammatory Index; eDII, energy-adjusted Dietary Inflammatory Index; RR, relative risk; HR, hazard ratio; CI, confidence interval.

A comparison of the roles of prediagnostic and postdiagnostic intake in terms of all-cause mortality is presented in Figure 3. Unhealthy prediagnostic and postdiagnostic patterns (RR/HR = 1.33, 95% CI = 1.09–1.62 and RR/HR = 1.47, 95% CI = 1.05–2.05, respectively), the insulin index (RR/HR = 1.32, 95% CI = 1.02–1.71 and RR/HR = 1.89, 95% CI = 1.22–2.91, respectively), and insulin load (RR/HR = 1.33, 95% CI = 1.03–1.72 and RR/HR = 2.30, 95% CI = 1.36–3.87, respectively) were consistently associated with an increased risk of all-cause mortality. Additionally, the prediagnostic and postdiagnostic ACS scores were consistently associated with a decreased risk of all-cause mortality, with RRs/HRs (95% CIs) of 0.78 (0.69–0.94) and 0.62 (0.43–0.89), respectively. The all-cause mortality was 73% lower in the participants with a high consumption of prediagnostic proteins (RR/HR = 0.27, 95% CI = 0.12–0.63) but 24% higher in those with high consumption of postdiagnostic proteins (RR/HR = 1.24, 95% CI = 1.03–1.49). The prediagnostic, but not postdiagnostic, intake of carbohydrates, proteins, lipids, saturated fatty acids (SFAs), and monounsaturated fatty acids (MUFAs) and the (modified and/or alternative) Mediterranean Diet score (MED/aMED) were associated with decreased risks of all-cause mortality, with RRs/HRs (95% CIs) of 0.32 (0.14–0.76), 0.27 (0.12–0.63), 0.24 (0.09–0.59), 0.20 (0.08–0.49), 0.35 (0.15–0.78), and 0.62 (0.39–0.98), respectively. In contrast, no association between the prediagnostic intake of milk, polyunsaturated fatty acid (PUFA), and fiber, Healthy Eating Index/alternate HEI (HEI/aHEI), or Dietary Inflammatory Index (DII/eDII) intake and all-cause mortality was observed, but significant associations between these parameters were observed for postdiagnostic intake, with RR/HR (95% CI) of 0.72 (0.55–0.94), 0.81 (0.66–0.98), 0.64 (0.51–0.80), 0.71 (0.52–0.97), and 1.37 (1.01–1.87), respectively.

**Figure 4:**
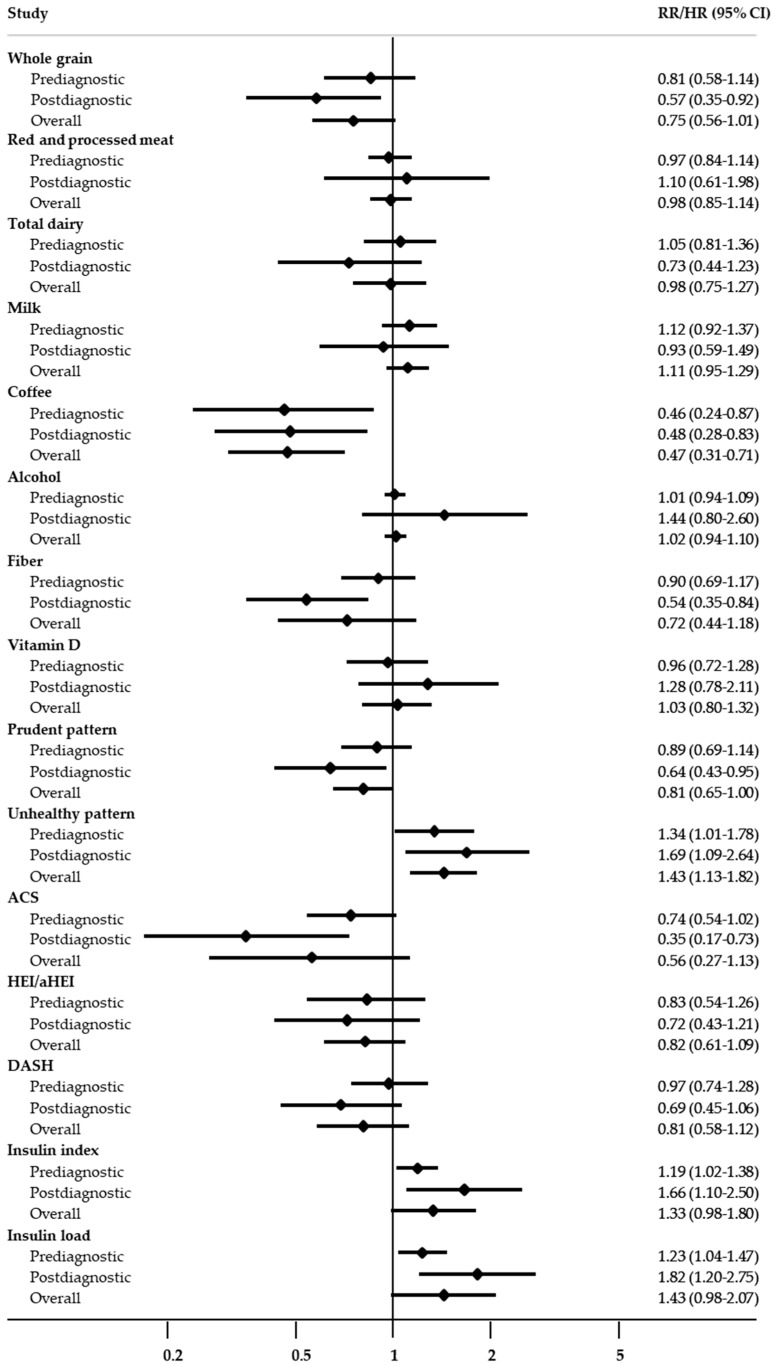
Comparison of the roles of prediagnostic and postdiagnostic intake in colorectal cancer-specific mortality. ACS, American Cancer Society recommendations score; DASH, Dietary Approaches to Stop Hypertension score; RR, relative risk; HR, hazard ratio; CI, confidence interval.

A comparison of the roles of prediagnostic and postdiagnostic intake in terms of CRC-specific mortality is presented in Figure 4. Consistently, significant associations were observed between the prediagnostic and postdiagnostic intake and coffee intake (RR/HR = 0.46, 95% CI = 0.24–0.87 and RR/HR = 0.48, 95% CI = 0.28–0.83, respectively), an unhealthy pattern (RR/HR = 1.34, 95% CI = 1.01–1.78 and RR/HR = 1.69, 95% CI = 1.09–2.64, respectively), the insulin index (RR/HR = 1.19, 95% CI = 1.02–1.38 and RR/HR = 1.66, 95% CI = 1.10–2.50, respectively), and insulin load (RR/HR = 1.23, 95% CI = 1.04–1.47 and RR/HR = 1.82, 95% CI = 1.20–2.75, respectively). Additionally, the postdiagnostic intake of whole grain, fiber, a prudent pattern, ACS score, and an unhealthy dietary pattern were significantly associated with CRC-specific mortality, with RRs/HRs (95% CIs) of 0.57 (0.35–0.92), 0.54 (0.35–0.84), 0.64 (0.43–0.95), 0.35 (0.17–0.73), and 1.69 (1.09–2.64), respectively; however, these associations were not observed with the prediagnostic intake. Instead, grilled food and the WCRF/AICR score were associated with CRC-specific mortality (RR/HR = 1.78, 95% CI = 1.05–3.02 and RR/HR = 0.70, 95% CI = 0.56–0.89) only before the CRC diagnosis.

## 4. Discussion

The current systematic review and meta-analysis of 45 prospective studies investigated the association between dietary intake and the risk of all-cause mortality and CRC-specific mortality among CRC survivors. Our findings indicated diverse patterns of the associations between all-cause mortality or CRC-specific mortality and each food item, nutrient, dietary pattern, or index. Additionally, similar associations with mortality before and after the CRC diagnosis were observed with red and processed meat, sugar-containing and sweet products, total dairy, coffee, alcohol, calcium, vitamin D, an unhealthy pattern, Dietary Approaches to Stop Hypertension (DASH) score, and insulin index or load among CRC survivors; however, different associations were found with the intake of whole grain, milk, the macronutrients of carbohydrates, proteins, lipids, SFA, MUFA, PUFA, and fiber intake; a prudent pattern; and dietary indexes, such as the ACS, HEI/aHEI, MED/aMED, and DII/eDII.

In this study, the risk of all-cause mortality varied by food item, nutrient, dietary pattern, and index. A significantly reduced risk of all-cause mortality was associated with the highest intake of fruits, whole grains, dark fish, coffee, and calcium and the lowest ACS score, while an unhealthy pattern and the highest insulin index were associated with an increased risk of all-cause mortality. Regarding CRC-specific mortality, coffee intake and an unhealthy dietary pattern were associated with a lower and higher risk of CRC-specific mortality, respectively. Our results are consistent with the findings of recent meta-analyses of prospective studies; inverse associations were observed between all-cause mortality and the intake of whole grains [61,62], fruit [62,63], fish [62], coffee [64], and calcium [65], and mostly nonlinear relationships were observed. However, in contrast to our results, in the previous meta-analyses, there were negative associations between all-cause mortality and vegetable [62,63] or nut [62] consumption; the HEI, aHEI, and DASH scores [66]; and a prudent/healthy dietary pattern [67]. Additionally, there was a positive association between all-cause mortality and red and processed meat intake [62] but no association with soy consumption [68] or a Western/unhealthy dietary pattern [67]. Of these dietary components, patterns, and indexes, a high intake of total calcium, dietary fiber, whole grains, and soy and the combination of the three indexes (HEI, aHEI, and DASH) were associated with a reduced risk of CRC [66,68,69,70,71]. Furthermore, a Western dietary pattern was associated with an increased risk of CRC-specific mortality [7]. Specifically, two previous meta-analyses of dietary factors and CRC-specific mortality were targeted to cancer survivors [7,66]; a Western dietary pattern had an adverse effect on CRC-specific mortality (RR = 1.55, 95% CI = 1.13–2.13, I^2^ = 35%), but no association was found with dairy or meat consumption or a prudent/healthy dietary pattern among 209,597 cancer survivors [7]. However, all three indexes of dietary quality combined (HEI, aHEI, and DASH) had a protective effect on CRC-specific mortality in a previous meta-analysis of seven studies involving cancer survivors (RR = 0.77, 95% CI = 0.73–0.81, I^2^ = 0%) [7]. Nevertheless, no specific information on guidelines regarding various dietary factors are available to date for overall cancer survivors, including CRC survivors. Concerning different intervention strategies that would be needed according to the cancer type, dietary recommendations for patients with CRC should be considered at the time of diagnosis.

Changes in health-related behaviors after diagnosis and treatment, such as dietary patterns or physical activity, have been investigated among CRC survivors. Van Zutphen et al. [4] reported that CRC survivors consumed significantly fewer sugary drinks (−45 g/day) and less red and processed meat (−62 g/week) at 2 years after diagnosis. In this study, similar patterns of the risk of CRC-specific mortality, rather than all-cause mortality, were significantly shown with prediagnostic and postdiagnostic dietary intake among CRC survivors, including coffee consumption, insulin index, and insulin load. The highest intake of coffee was associated with a lower risk of CRC-specific mortality both before and after the CRC diagnosis. However, the highest insulin index or load group was found to have a higher risk of CRC-specific mortality. Consistent with our results, the Nurses’ Health Study and Health Professional Follow-up Study found that 1599 CRC patients with a stage of I to III who maintained coffee intake more than two cups a day after the CRC diagnosis had a lower risk of CRC-specific mortality (HR = 0.63, 95% CI = 0.44–0.89) than those consuming coffee intake below two cups a day before and after diagnosis [27]. A slightly weaker inverse association between coffee intake and all-cause mortality has been observed (HR = 0.71, 95% CI = 0.60–0.85; maintaining ≥ 2 cups/day vs. maintaining < 2 cups/day) [27]. Interestingly, in a subgroup analysis of the insulin load or index, which were defined as risk factors for both all-cause mortality and CRC-specific mortality in this study, a lower risk of CRC-specific mortality with coffee intake was observed only in the lower category of insulin load (HR = 0.83, 95% CI = 0.70–0.98) despite the null association for the dietary insulin index [27]. The insulin index can be less reflective of the long-term effect on CRC-specific mortality than the insulin load [72], and its role in CRC-specific mortality among survivors may result from the combination of dietary items they consumed, such as carbohydrates, proteins, and fiber, or the strongest item among these dietary components.

In this study, the all-cause mortality and CRC-specific mortality were consistently and positively affected by an unhealthy dietary pattern depending on the CRC prediagnostic and postdiagnosis status. Regarding the prediagnostic unhealthy dietary patterns, which were defined as a high-sugar or processed meat patterns, there were significant positive associations with all-cause mortality (RR = 1.33, 95% CI = 1.09–1.62) and CRC-specific mortality (RR = 1.34, 95% CI = 1.01–1.78). Furthermore, regarding the postdiagnostic unhealthy patterns, namely, the Western dietary pattern reported in three studies, significant positive associations were observed with both all-cause mortality (RR = 1.47, 95% CI = 1.05–2.05) and CRC-specific mortality (RR = 1.69, 95% CI = 1.09–2.64). These results could be attributable to the combination of dietary factors comprising the unhealthy dietary pattern, rather than each dietary component; however, little knowledge regarding its definition among CRC survivors is limited. On the basis of the three articles included in the final analysis, an unhealthy pattern was commonly characterized by high intake of red and processed meats and refined grains, as well as additionally included sweets, desserts, and high-fat dairy products [4]; eggs, solid fats, and salty snacks [4]; or desserts, high-fat dairy products, and French fries [4]. Further studies are needed to investigate the effect of a Western dietary pattern on mortality on the basis of a clarified combination of dietary components.

A large prospective study of 31,456 deaths during 9 years of follow-up observed the protective effect of dietary fiber intake on reducing all-cause mortality among both men (RR = 0.78, 95% CI = 0.73–0.82) and women (RR = 0.78, 95% CI = 0.73–0.85) [73], which was consistent with our findings of postdiagnostic fiber intake. However, fiber intake was associated with a decreased risk of cancer death among men only (RR = 0.83, 95% CI = 0.76–0.92) and not among women (RR = 0.96, 95% CI = 0.85–1.08) [73]. In contrast, prediagnostic fiber intake was consistently not associated with all-cause mortality or CRC-specific mortality among CRC survivors [20,50,56]. Although the amount of prediagnostic and postdiagnostic fiber consumption did not differ among Norwegian women with a CRC diagnosis [50,74], a per 5 g/day increase in fiber intake after the CRC diagnosis was associated with 14% and 18% decreased risks of all-cause mortality and CRC-specific mortality, respectively [44]. However, the role of carbohydrates in the risk of CRC has been controversial [75,76,77,78]. A pooled analysis of 17 observational studies found that a high consumption of carbohydrates was not associated with CRC and that it did not differ by colon or rectal cancer [75]. Despite the small number of individual studies in our analysis, a significant positive association was observed in the effect of carbohydrate intake on CRC-specific mortality, but not all-cause mortality, leading to the result of postdiagnostic carbohydrate intake. Considering that a high intake of carbohydrates could result in a higher dietary insulin index and load [72], further studies are warranted to specify the effects on CRC-specific mortality by the source of carbohydrate intake following the CRC diagnosis. There has also been limited evidence regarding the role of protein intake in CRC-specific mortality, and only one null association has been reported thus far [79]. Therefore, changes in many dietary factors before and after a disease diagnosis [80] may represent an important factor contributing to mortality outcomes. A recent systematic review of diet and physical activity related to the quality of life of CRC survivors highlighted the importance of a healthy diet along with physical activity for reducing the risk of recurrence [81], suggesting adherence to a healthy lifestyle is necessary for patients with CRC to improve their quality of life and prolong their lives. Regarding a prudent pattern, null associations between all-cause mortality and both the CRC prediagnostic and postdiagnosis intake were observed. A protective effect on CRC-specific mortality was observed only with a postdiagnostic prudent pattern consumption.

To the best of our knowledge, this is the first study to analyze all the available data of both prediagnostic and postdiagnostic dietary intake in association with all-cause mortality and CRC-specific mortality among CRC survivors. The current systematic review and meta-analysis included only prospective studies, which are assumed to have a higher level of evidence and are less susceptible to recall bias and selection bias than retrospective studies [82]. Additionally, the methodological quality assessment showed that all the studies included in the final analysis were of high quality. Furthermore, in all studies concerning dietary intake and mortality outcomes, adjustments were made for major risk factors, suggesting that the findings were more reliable. Despite its strengths, this study has some limitations. First, the association between several dietary items and the mortality outcomes was reported from a single study only, which did not allow us to calculate the pooled estimate of various study populations. Second, the cut-off level of the highest and lowest quantiles was generally unclear and nonuniform across the individual studies, suggesting that both overestimations and underestimations could have occurred [83]. Third, the intrinsic limitation of potential biases from individual studies included in the final analysis might affect our pooled estimates. Whether the dietary habits observed at baseline were maintained during follow-up was unclear. The subjects who were aware of the important role of a healthy diet might change their consumption behavior after the cancer diagnosis [84]. Finally, the dietary intake in the included studies was obtained by using an FFQ, which is completed by the participants themselves or via an interview [82]. Both web-based and print-based FFQ have been reported to share measurement error [85], which may affect the power of detecting the diet-mortality association.

## 5. Conclusions

In summary, this study provided comprehensive evidence of the effect of all prediagnostic and postdiagnostic dietary items on mortality outcomes. Overall, unhealthy consumption patterns, including a Western diet, the intake of processed meat, or high-sugar dietary patterns, was found to increase the risks of both all-cause mortality and CRC-specific mortality. Prediagnostic and postdiagnostic intake of whole grain, carbohydrates, proteins, lipids, SFAs, MUFAs, PUFAs, and fiber played different roles in the risk of all-cause mortality.

## Figures and Tables

**Table 1:** Systematic review and meta-analysis of the associations between overall dietary intake and mortality among colorectal cancer survivors.

Dietary Factor	All-Cause Mortality		CRC Mortality	
	*N* (I^2^)	RR/HR (95% CI)	*N* (I^2^)	RR/HR (95% CI)
Food items				
Fruits	2 (0%)	**0.88 (0.80–0.96)**	2 (64%)	0.80 (0.52–1.24)
Vegetables	2 (0%)	0.92 (0.84–1.01)	1 (NA)	1.10 (0.82–1.47)
Whole grain	3 (0%)	**0.83 (0.69–0.99)**	4 (46%)	0.75 (0.56–1.01)
Refined grain	1 (NA)	**1.88 (1.25–2.85)**		
Wheat	2 (0%)	1.08 (0.76–1.53)		
Rye	2 (0%)	0.91 (0.68–1.23)		
Oats	2 (0%)	0.96 (0.71–1.29)		
Meat	1 (NA)	1.64 (0.75–3.58)	1 (NA)	0.97 (0.85–1.10)
Poultry	1 (NA)	0.87 (0.73–1.03)	1 (NA)	0.91 (0.75–1.10)
Red and processed meat	4 (59.0%)	1.02 (0.85–1.22)	4 (0%)	0.98 (0.85–1.14)
Red meat	3 (0%)	1.01 (0.91–1.12)	4 (52.4%)	1.06 (0.89–1.27)
Processed meat	3 (0%)	1.02 (0.95–1.10)	3 (0%)	1.07 (0.94–1.23)
Fish	1 (NA)	1.24 (0.58–2.65)		
Dark fish	1 (NA)	0.68 (0.48–0.96)		
Canned tuna fish	1 (NA)	1.23 (0.82–1.84)		
Other fish	1 (NA)	1.03 (0.71–1.48)		
Fish products	1 (NA)	1.10 (0.74–1.64)		
Other seafood	1 (NA)	1.04 (0.73–1.50)		
Eggs	1 (NA)	1.10 (0.52–2.33)		
Grilled food			1 (NA)	**1.78 (1.05–3.02)**
Rice and noodle	1 (NA)	0.97 (0.44–2.15)		
Bread	1 (NA)	1.01 (0.43–2.33)		
Sugar and sweet product	2 (0%)	1.09 (0.80–1.50)		
Total dairy	4 (66.8%)	0.90 (0.71–1.15)	3 (51.3%)	0.98 (0.75–1.27)
Milk	3 (82.1%)	0.95 (0.72–1.26)	3 (0%)	1.11 (0.95–1.29)
Cheese	1 (NA)	0.87 (0.74–1.04)	1 (NA)	
Yogurt	1 (NA)	1.08 (0.92–1.28)	1 (NA)	
Tea and coffee	1 (NA)	1.46 (0.65–3.27)		
Tea	1 (NA)	0.82 (0.40–1.68)		
Coffee	2 (0%)	**0.69 (0.55–0.98)**	2 (0%)	**0.47 (0.31–0.71)**
Sugar-sweetened beverage	2 (86.4%)	0.84 (0.32–2.23)		
Alcohol	6 (48.1%)	1.01 (0.87–1.16)	6 (0%)	1.02 (0.94–1.10)
Beer	1 (NA)	0.93 (0.80–1.08)	3 (0%)	1.01 (0.86–1.20)
Wine	1 (NA)	0.81 (0.65–1.02)	3 (0%)	0.83 (0.64–1.08)
Liquor	1 (NA)	0.89 (0.73–1.08)	3 (0%)	0.92 (0.75–1.13)
Macronutrients				
Carbohydrates	3 (85.6%)	1.02 (0.55–1.90)	1 (NA)	**1.91 (1.17–3.12)**
Proteins	2 (91.9%)	0.61 (0.14–2.71)	1 (NA)	1.01 (0.70–1.46)
Lipids	2 (85.5%)	0.50 (0.14–1.74)	1 (NA)	0.68 (0.44–1.06)
SFA	2 (90.3%)	0.46 (0.10–2.06)	1 (NA)	1.30 (0.77–2.19)
MUFA	2 (77.8%)	0.61 (0.25–1.49)	1 (NA)	0.87 (0.50–1.53)
PUFA	2 (22.1%)	0.75 (0.55–1.04)	1 (NA)	0.72 (0.48–1.09)
Omega-3	3 (0%)	0.91 (0.77–1.07)		
Fiber	3 (73.8%)	0.84 (0.58–1.22)	2 (73.4%)	0.72 (0.44–1.18)
Micronutrients				
Calcium	4 (0%)	**0.84 (0.73–0.97)**	3 (22.3%)	0.82 (0.62–1.07)
Iron	1 (NA)	0.56 (0.21–1.46)		
Copper	1 (NA)	0.59 (0.26–1.34)		
Zinc	1 (NA)	0.92 (0.38–2.23)		
Vitamin A	1 (NA)	1.43 (0.71–2.88)		
Beta-carotene	1 (NA)	1.59 (0.74–3.43)		
Vitamin B1	1 (NA)	0.75 (0.29–1.95)		
Vitamin B2	1 (NA)	0.70 (0.31–1.59)		
Vitamin B6	1 (NA)	1.02 (0.45–1.17)		
Folic acid	1 (NA)	1.67 (0.74–3.78)		
Vitamin B12	1 (NA)	1.53 (0.73–3.21)		
Vitamin C	1 (NA)	1.11 (0.50–2.49)		
Vitamin D	3 (0%)	0.97 (0.83–1.13)	2 (0%)	1.03 (0.80–1.32)
Vitamin E	1 (NA)	1.43 (0.62–3.31)		
Vitamin PP	1 (NA)	1.15 (0.45–2.91)		
Pantothenic acid	1 (NA)	1.04 (0.44–2.44)		
Biotine	1 (NA)	1.12 (0.50–2.50)		
Total flavonoids	1 (NA)	0.97 (0.60–1.56)	1 (NA)	0.87 (0.47–1.62)
Flavanols	1 (NA)	0.99 (0.63–1.58)	1 (NA)	1.34 (0.73–2.45)
Flavan-3-ol monomers	1 (NA)	0.93 (0.59–1.46)	1 (NA)	0.91 (0.49–1.67)
Proanthocyanidins	1 (NA)	1.08 (0.68–1.71)	1 (NA)	1.30 (0.71–2.39)
Flavonols	1 (NA)	0.90 (0.58–1.39)	1 (NA)	1.18 (0.65–2.13)
Flavanones	1 (NA)	0.92 (0.60–1.42)	1 (NA)	0.80 (0.46–1.39)
Anthocyanidins	1 (NA)	0.91 (0.58–1.44)	1 (NA)	0.87 (0.48–1.57)
Flavones	1 (NA)	0.87 (0.56–1.36)	1 (NA)	0.97 (0.54–1.73)
Isoflavones	1 (NA)	0.97 (0.62–1.53)	1 (NA)	0.60 (0.33–1.09)
Lignans	1 (NA)	0.83 (0.50–1.37)	1 (NA)	0.68 (0.36–1.26)
Dietary patterns				
Prudent pattern	5 (0%)	**0.90 (0.83–0.97)**	4 (0%)	0.81 (0.65–1.00)
Unhealthy pattern	6 (0%)	**1.36 (1.17–1.57)**	5 (0%)	**1.43 (1.13–1.82)**
Dietary index				
ACS	3 (46.3%)	**0.70 (0.56–0.86)**	2 (70.5%)	0.56 (0.27–1.13)
HEI/aHEI	3 (65.7%)	0.76 (0.58–1.01)	3 (41.1%)	0.82 (0.61–1.09)
MED/aMED	2 (28.8%)	0.76 (0.55–1.05)	1 (NA)	0.84 (0.50–1.42)
DASH	3 (0%)	0.89 (0.79–1.01)	3 (49.6%)	0.81 (0.58–1.12)
DII/eDII	3 (57.8%)	1.15 (0.81–1.65)		
Glycemic index	1 (NA)	1.23 (0.83–1.82)	1 (NA)	1.02 (0.89–1.16)
Glycemic load	1 (NA)	**1.74 (1.20–2.51)**	1 (NA)	1.10 (0.94–1.29)
Insulin index	2 (48.3%)	**1.51 (1.07–2.12)**	2 (55.0%)	1.33 (0.98–1.80)
Insulin load	2 (70.6%)	1.66 (0.98–2.82)	2 (65.8%)	1.43 (0.98–2.07)
HNFI	1 (NA)	0.63 (0.39–1.04)		
WCRF/AICR score	1 (NA)	**0.79 (0.65–0.98)**	1 (NA)	**0.70 (0.56–0.89)**
Recommended food score	1 (NA)	0.65 (0.60–1.67)		
Diet quality score			1 (NA)	0.63 (0.32–1.21)

*N*, number of individual studies; NA, not applicable; SFA, saturated fatty acids; MUFA, monounsaturated fatty acids; PUFA, polyunsaturated fatty acids; ACS, American Cancer Society recommendations score; HEI, Healthy Eating Index; aHEI, alternate Healthy Eating Index; MED, Mediterranean Diet score; aMED, alternate Mediterranean Diet score; DASH, Dietary Approaches to Stop Hypertension score; DII, Dietary Inflammatory Index; eDII, energy-adjusted Dietary Inflammatory Index; HNFI, Healthy Nordic Food Index; WCRF/AICR, World Cancer Research Fund/American Institute of Cancer Research. Bold font indicates statistical significance.

**Table 2:** Systematic review and meta-analysis of the associations between prediagnostic dietary intake and mortality among colorectal cancer survivors.

Dietary Factor	All-Cause Mortality		CRC Mortality	
	*N* (I^2^)	RR/HR (95% CI)	*N* (I^2^)	RR/HR (95% CI)
Food items				
Fruits	2 (0%)	**0.88 (0.80–0.96)**	2 (64%)	0.80 (0.52–1.24)
Vegetables	2 (0%)	0.92 (0.84–1.01)	1 (NA)	1.10 (0.82–1.47)
Whole grain			3 (46.8%)	0.81 (0.58–1.14)
Wheat	2 (0%)	1.08 (0.76–1.53)		
Rye	2 (0%)	0.91 (0.68–1.23)		
Oats	2 (0%)	0.96 (0.71–1.29)		
Meat	1 (NA)	1.64 (0.75–3.58)	1 (NA)	0.97 (0.85–1.10)
Poultry	1 (NA)	0.87 (0.73–1.03)	1 (NA)	0.91 (0.75–1.10)
Red and processed meat	3 (71.2%)	1.04 (0.83–1.30)	3 (0%)	0.97 (0.84–1.14)
Red meat	3 (0%)	1.01 (0.91–1.12)	4 (52.4%)	1.06 (0.89–1.27)
Processed meat	3 (0%)	1.02 (0.95–1.10)	3 (0%)	1.07 (0.94–1.23)
Fish	1 (NA)	1.24 (0.58–2.65)		
Eggs	1 (NA)	1.10 (0.52–2.33)		
Grilled food			1 (NA)	**1.78 (1.05–3.02)**
Rice and noodle	1 (NA)	0.97 (0.44–2.15)		
Bread	1 (NA)	1.01 (0.43–2.33)		
Sugar and sweet product	1 (NA)	1.02 (0.48–2.16)		
Total dairy	3 (66.2%)	0.97 (0.74–1.26)	2 (51.7%)	1.05 (0.81–1.36)
Milk	2 (72.3%)	1.08 (0.85–1.36)	2 (25.0%)	1.12 (0.92–1.37)
Cheese	1 (NA)	0.87 (0.74–1.04)	1 (NA)	0.93 (0.76–1.14)
Yogurt	1 (NA)	1.08 (0.92–1.28)	1 (NA)	1.09 (0.88–1.34)
Tea and coffee	1 (NA)	1.46 (0.65–3.27)		
Coffee			1 (NA)	0.46 (0.24–0.87)
Alcohol	5 (57.6%)	1.02 (0.87–1.21)	5 (0%)	1.01 (0.94–1.09)
Beer	1 (NA)	0.93 (0.80–1.08)	3 (0%)	1.01 (0.86–1.20)
Wine	1 (NA)	0.81 (0.65–1.02)	3 (0%)	0.83 (0.64–1.08)
Liquor	1 (NA)	0.89 (0.73–1.08)	3 (0%)	0.92 (0.75–1.13)
Macronutrients				
Carbohydrates	1 (NA)	**0.32 (0.14–0.76)**		
Proteins	1 (NA)	**0.27 (0.12–0.63)**		
Lipids	1 (NA)	**0.24 (0.09–0.59)**		
SFA	1 (NA)	**0.20 (0.08–0.49)**		
MUFA	1 (NA)	**0.35 (0.15–0.78)**		
PUFA	1 (NA)	0.52 (0.25–1.10)		
Omega-3	1 (NA)	0.93 (0.70–1.23)		
Fiber	2 (70.8%)	1.14 (0.53–2.43)	1 (NA)	0.90 (0.69–1.17)
Micronutrients				
Calcium	2 (0%)	0.85 (0.71–1.03)		
Iron	1 (NA)	0.56 (0.21–1.46)		
Copper	1 (NA)	0.59 (0.26–1.34)		
Zinc	1 (NA)	0.92 (0.38–2.23)		
Vitamin A	1 (NA)	1.43 (0.71–2.88)		
Beta-carotene	1 (NA)	1.59 (0.74–3.43)		
Vitamin B1	1 (NA)	0.75 (0.29–1.95)		
Vitamin B2	1 (NA)	0.70 (0.31–1.59)		
Vitamin B6	1 (NA)	1.02 (0.45–1.17)		
Folic acid	1 (NA)	1.67 (0.74–3.78)		
Vitamin B12	1 (NA)	1.53 (0.73–3.21)		
Vitamin C	1 (NA)	1.11 (0.50–2.49)		
Vitamin D	2 (19.2%)	1.03 (0.77–1.38)	1 (NA)	0.96 (0.72–1.28)
Vitamin E	1 (NA)	1.43 (0.62–3.31)		
Vitamin PP	1 (NA)	1.15 (0.45–2.91)		
Pantothenic acid	1 (NA)	1.04 (0.44–2.44)		
Biotine	1 (NA)	1.12 (0.50–2.50)		
Total flavonoids	1 (NA)	0.97 (0.60–1.56)	1 (NA)	0.87 (0.47–1.62)
Flavanols	1 (NA)	0.99 (0.63–1.58)	1 (NA)	1.34 (0.73–2.45)
Flavan-3-ol monomers	1 (NA)	0.93 (0.59–1.46)	1 (NA)	0.91 (0.49–1.67)
Proanthocyanidins	1 (NA)	1.08 (0.68–1.71)	1 (NA)	1.30 (0.71–2.39)
Flavonols	1 (NA)	0.90 (0.58–1.39)	1 (NA)	1.18 (0.65–2.13)
Flavanones	1 (NA)	0.92 (0.60–1.42)	1 (NA)	0.80 (0.46–1.39)
Anthocyanidins	1 (NA)	0.91 (0.58–1.44)	1 (NA)	0.87 (0.48–1.57)
Flavones	1 (NA)	0.87 (0.56–1.36)	1 (NA)	0.97 (0.54–1.73)
Isoflavones	1 (NA)	0.97 (0.62–1.53)	1 (NA)	0.60 (0.33–1.09)
Lignans	1 (NA)	0.83 (0.50–1.37)	1 (NA)	0.68 (0.36–1.26)
Dietary patterns				
Prudent pattern	2 (0%)	0.86 (0.74–1.00)	2 (0%)	0.89 (0.69–1.14)
Unhealthy pattern	3 (0%)	**1.33 (1.09–1.62)**	3 (0%)	**1.34 (1.01–1.78)**
Dietary index				
ACS	1 (NA)	**0.78 (0.65–0.94)**	1 (NA)	0.74 (0.54–1.02)
HEI/aHEI	2 (79.3%)	0.77 (0.49–1.21)	2 (64.9%)	0.83 (0.54–1.26)
MED/aMED	1 (NA)	**0.62 (0.39–0.98)**		
DASH	1 (NA)	0.92 (0.78–1.08)	1 (NA)	0.97 (0.74–1.28)
DII/eDII	1 (NA)	0.78 (0.48–1.27)		
Glycemic index			1 (NA)	1.02 (0.89–1.16)
Glycemic load			1 (NA)	1.10 (0.94–1.29)
Insulin index	1 (NA)	**1.32 (1.02–1.71)**	1 (NA)	**1.19 (1.02–1.38)**
Insulin load	1 (NA)	**1.33 (1.03–1.72)**	1 (NA)	**1.23 (1.04–1.47)**
HNFI	1 (NA)	0.63 (0.39–1.04)		
WCRF/AICR score	1 (NA)	**0.79 (0.65–0.98)**	1 (NA)	**0.70 (0.56–0.89)**
Recommended food score	1 (NA)	1.54 (0.92–2.56)		
Diet quality score			1 (NA)	0.63 (0.32–1.21)

*N*, number of individual studies; NA, not applicable; SFA, saturated fatty acids; MUFA, monounsaturated fatty acids; PUFA, polyunsaturated fatty acids; HEI, Healthy Eating Index; aHEI, alternate Healthy Eating Index; MED, Mediterranean Diet score; aMED, alternate Mediterranean Diet score; DII, Dietary Inflammatory Index; eDII, energy-adjusted Dietary Inflammatory Index; HNFI, Healthy Nordic Food Index; WCRF/AICR, World Cancer Research Fund/American Institute of Cancer Research. Bold font indicates statistical significance.

**Table 3:** Systematic review and meta-analysis of the associations between postdiagnostic dietary intake and mortality among colorectal cancer survivors.

Dietary Factor	All-Cause Mortality		CRC Mortality	
	*N* (I^2^)	RR/HR (95% CI)	*N* (I^2^)	RR/HR (95% CI)
Food items				
Whole grain	3 (0%)	**0.83 (0.69–0.99)**	1 (NA)	**0.57 (0.35–0.92)**
Refined grain	1 (NA)	**1.88 (1.25–2.85)**		
Red and processed meat	1 (NA)	0.94 (0.68–1.30)	1 (NA)	1.10 (0.61–1.98)
Dark fish	1 (NA)	**0.68 (0.48–0.96)**		
Canned tuna fish	1 (NA)	1.23 (0.82–1.84)		
Other fish	1 (NA)	1.03 (0.71–1.48)		
Fish products	1 (NA)	1.10 (0.74–1.64)		
Other seafood		1.04 (0.73–1.50)		
Sugar and sweet products	1 (NA)	1.11 (0.79–1.58)		
Total dairy	1 (NA)	0.75 (0.56–1.01)	1 (NA)	0.73 (0.44–1.23)
Milk	1 (NA)	**0.72 (0.55–0.94)**	1 (NA)	0.93 (0.59–1.49)
Tea	1 (NA)	0.82 (0.40–1.68)		
Coffee	2 (0%)	**0.69 (0.55–0.98)**	1 (NA)	**0.48 (0.28–0.83)**
Sugar-sweetened beverages	2 (86.4%)	0.84 (0.32–2.23)		
Alcohol	1 (NA)	0.92 (0.66–1.28)	1 (NA)	1.44 (0.80–2.60)
Macronutrients				
Carbohydrates	2 (69.5%)	1.44 (0.98–2.11)	1 (NA)	**1.91 (1.17–3.12)**
Proteins	1 (NA)	**1.24 (1.03–1.49)**	1 (NA)	1.01 (0.70–1.46)
Lipids	1 (NA)	0.87 (0.72–1.07)	1 (NA)	0.68 (0.44–1.06)
SFA	1 (NA)	0.93 (0.74–1.18)	1 (NA)	1.30 (0.77–2.19)
MUFA	1 (NA)	0.89 (0.69–1.14)	1 (NA)	0.87 (0.50–1.53)
PUFA	1 (NA)	0.81 (0.66–0.98)	1 (NA)	0.72 (0.48–1.09)
Omega-3	2 (0%)	0.89 (0.72–1.10)		
Fiber	1 (NA)	0.64 (0.51–0.80)	1 (NA)	**0.54 (0.35–0.84)**
Micronutrients				
Calcium	2 (0%)	0.83 (0.68–1.02)	2 (57.9%)	0.76 (0.43–1.34)
Vitamin D	1 (NA)	0.90 (0.67–1.21)	1 (NA)	1.28 (0.78–2.11)
Dietary patterns				
Prudent pattern	3 (34.8%)	0.95 (0.79–1.15)	2 (0%)	**0.64 (0.43–0.95)**
Unhealthy pattern	3 (52.9%)	**1.47 (1.05–2.05)**	2 (0%)	**1.69 (1.09–2.64)**
Dietary index				
ACS	2 (55.8%)	**0.62 (0.43–0.89)**	1 (NA)	**0.35 (0.17–0.73)**
HEI/aHEI	1 (NA)	**0.71 (0.52–0.97)**	1 (NA)	0.72 (0.43–1.21)
MED/aMED	1 (NA)	0.87 (0.63–1.21)	1 (NA)	0.84 (0.50–1.42)
DASH	2 (11.5%)	0.85 (0.70–1.05)	2 (35.5%)	0.69 (0.45–1.06)
DII/eDII	2 (0%)	**1.37 (1.01–1.87)**		
Glycemic index	1 (NA)	1.23 (0.83–1.82)		
Glycemic load	1 (NA)	**1.74 (1.20–2.51)**		
Insulin index	1 (NA)	**1.89 (1.22–2.91)**	1 (NA)	**1.66 (1.10–2.50)**
Insulin load	1 (NA)	**2.30 (1.36–3.87)**	1 (NA)	**1.82 (1.20–2.75)**

*N*, number of individual studies; NA, not applicable; SFA, saturated fatty acids; MUFA, monounsaturated fatty acids; PUFA, polyunsaturated fatty acids; ACS, American Cancer Society recommendations score; HEI, Healthy Eating Index; aHEI, alternate Healthy Eating Index; MED, Mediterranean Diet score; aMED, alternate Mediterranean Diet score; DASH, Dietary Approaches to Stop Hypertension score; DII, Dietary Inflammatory Index; eDII, energy-adjusted Dietary Inflammatory Index. Bold font indicates statistical significance.

## Supplementary Materials

The following are available online at https://www.mdpi.com/2072-6694/12/11/3391/s1: Table S1: Search strategy. Table S2: Inclusion and exclusion criteria. Table S3: Baseline characteristics of the studies included in the final systematic review and meta-analysis. Table S4: Newcastle–Ottawa quality assessment scale of the cohort studies included in the systematic review and meta-analysis. Table S5: Assessment of attrition bias in the individual studies.
