# Supplementary material for: Dietary Intake in Association with All-Cause Mortality and Colorectal Cancer Mortality among Colorectal Cancer Survivors: A Systematic Review and Meta-Analysis of Prospective Studies
Source: Cancers (Basel). 2020 Nov 16;12(11):3391. doi: 10.3390/cancers12113391 (PMC7697273; doi:10.3390/cancers12113391)
Supplement: Supplementary file 1 [file cancers-12-03391-s001.pdf]

Dietary Intake in Association with All-Cause Mortality and Colorectal Cancer Mortality among Colorectal Cancer Survivors: A Systematic Review And Meta-Analysis of Prospective Studies

Tung Hoang Hyejin Kim and Jeongseon Kim

Table S1. Search strategy.

Set	Item	Field (PubMed)	Field (Embase)
1	Diet	[Mesh]	[Emtree]
2	Nutrition	[Mesh]	[Emtree]
3	Dietary	[Mesh]	[Emtree]
4	Consumption	[Mesh]	[Emtree]
5	Intake	[Mesh]	[Emtree]
6	Sets 1-5 were combined with "OR"		
7	Colorectal	[Mesh]	[Emtree]
8	Colon	[Mesh]	[Emtree]
9	Rectum	[Mesh]	[Emtree]
10	Rectal	[Mesh]	[Emtree]
11	Sets 7-10 were combined with "OR"		
12	Cancer	[Mesh]	[Emtree]
13	Survival	[Mesh]	[Emtree]
14	Mortality	[Mesh]	[Emtree]
15	Sets 13-14 were combined with "OR"		
16	Sets 6, 11, 12, 15 were combined with "AND"		

Syntax in PubMed: (("diet"[MeSH Terms] OR "diet"[All Fields] OR ("nutrition s"[All Fields] OR "nutritional status"[MeSH Terms] OR ("nutritional"[All Fields] AND "status"[All Fields]) OR "nutritional status"[All Fields] OR "nutrition"[All Fields] OR "nutritional sciences"[MeSH Terms] OR ("nutritional"[All Fields] AND "sciences"[All Fields]) OR "nutritional sciences"[All Fields] OR "nutritional"[All Fields] OR "nutritionals"[All Fields] OR "nutritions"[All Fields] OR "nutritive"[All Fields]) OR ("diet"[MeSH Terms] OR "diet"[All Fields] OR "dietary"[All Fields] OR "dietaries"[All Fields]) OR ("consumptions"[All Fields] OR "economics"[MeSH Terms] OR "economics"[All Fields] OR "consumption"[All Fields]) OR ("intake"[All Fields] OR "intake s"[All Fields] OR "intakes"[All Fields])) AND ("colorectal"[All Fields] OR ("colon"[MeSH Terms] OR "colon"[All Fields] OR "colonic"[All Fields] OR "colons"[All Fields] OR "colon s"[All Fields] OR "colonal"[All Fields] OR "colonically"[All Fields] OR "colonitis"[All Fields]) OR ("rectum"[MeSH Terms] OR "rectum"[All Fields] OR "rectums"[All Fields]) OR ("administration, rectal"[MeSH Terms] OR ("administration"[All Fields] AND "rectal"[All Fields]) OR "rectal administration"[All Fields] OR "rectal"[All Fields]))) AND ("cancer s"[All Fields] OR "cancerated"[All Fields] OR "canceration"[All Fields] OR "cancerization"[All Fields] OR "cancerized"[All Fields] OR "cancerous"[All Fields] OR "neoplasms"[MeSH Terms] OR "neoplasms"[All Fields] OR "cancer"[All Fields] OR "cancers"[All Fields]) AND ("mortality"[MeSH Subheading] OR "mortality"[All Fields] OR "survival"[All Fields] OR "survival"[MeSH Terms] OR "survivability"[All Fields] OR "survivable"[All Fields] OR "survivals"[All Fields] OR "survive"[All Fields] OR "survived"[All Fields] OR "survives"[All Fields] OR "surviving"[All Fields] OR ("mortality"[MeSH Terms] OR "mortality"[All Fields] OR "mortalities"[All Fields] OR "mortality"[MeSH Subheading])). Syntax in Embase: ('diet'/exp OR diet OR 'nutrition'/exp OR nutrition OR dietary OR 'consumption'/exp OR consumption OR intake) AND (colorectal OR 'colon'/exp OR colon OR 'rectum'/exp OR rectum OR rectal) AND ('cancer'/exp OR cancer) AND ('survival'/exp OR survival OR 'mortality'/exp OR mortality)

Table S2. Inclusion and exclusion criteria.

Factors	Inclusion criteria	Exclusion criteria
Study design	Cohort study or randomized controlled trial	In vitro, animal, or experimental study
Exposure	Any kind of dietary intake (single food items, nutrients, dietary patterns, or dietary indexes)	Unrelated exposure
Outcome	Mortality due to any cause, CRC, colon cancer, or rectal cancer	Unrelated outcome
Setting	Full-text version of the article was available	Abstract or review
Methodology	RR or HR and their 95% CIs	Studies with uncertain information about RR or HR

CRC, colorectal cancer; RR, relative risk; HR, hazard ratio; CIs, confidence intervals.

Table S3. Baseline characteristics of the studies included in the final systematic review and meta-analysis.

Study	Project, period (follow-up)	Diagnostic	Age (SD)	Sample size (male/ female)	Exposure	Outcome	RR/HR (95% CI)	Covariates
Brown 2018	CALGB 89803 (Alliance)	Postdiagnostic	60 [b]	574/ 444	Refined grain (≥3 vs. <1 servings/day)	All-cause mortality	1.88 (1.25–2.85)	Age, sex, race, performance status, T stage, positive lymph nodes, location of primary tumor, treatment arm, time-varying BMI, physical activity, total energy, and whole grain intake (for refined grain models) or refined grain intake (for whole grain models).
					Whole grain (≥3 vs. <1 servings/day)		0.86 (0.62–1.20)	
Carr 2016	DACHS	Prediagnostic	68 [a]	1,853/ 1,269	Red and processed meat (>1 vs. <1 time/day)	All-cause mortality	0.85 (0.67–1.09)	Age at diagnosis, sex, cancer stage, chemotherapy, surgery, BMI, physical activity, diabetes, stroke, heart failure, myocardial infarction, dairy intake, wholegrain intake, time between diagnosis and interview, and a time-dependent effect of chemotherapy 3 log(time).
					Red and processed meat (>1 vs. <1 time/day)	CRC-specific mortality	0.83 (0.61–1.14)	
Carr 2019	CRC Consortium (10 studies)	Prediagnostic	69 [a]	6,695 (male)	Red meat (Q4 vs. Q1, servings/day)	All-cause mortality	1.08 (0.93–1.26)	Age at diagnosis, sex, cancer site, BMI, smoking status, family history of CRC, aspirin/NSAIDs use, and education.
					Processed meat (Q4 vs. Q1, servings/ day)		1.10 (0.93–1.32)	
					Red meat (Q4 vs. Q1, servings/day)	CRC-specific mortality	1.09 (0.89–1.33)	
					Processed meat (Q4 vs. Q1, servings/ day)		1.11 (0.87–1.42)	

Study	Project, period (follow-up)	Diagnostic	Age (SD)	Sample size (male/ female)	Exposure	Outcome	RR/HR (95% CI)	Covariates
				930 (female)	Red meat (Q4 vs. Q1, servings/day)	All-cause mortality	0.95 (0.76–1.17)	
					Processed meat (Q4 vs. Q1, servings/ day)		0.98 (0.76–1.26)	
					Red meat (Q4 vs. Q1, servings/day)	CRC-specific mortality	0.96 (0.77–1.19)	
					Processed meat (Q4 vs. Q1, servings/ day)		0.98 (0.76–1.27)	
Dik 2014	EPIC	Prediagnostic	64.2 [a] (8.1)	3,859	Total dairy (Q4 vs. Q1, g/day)	All-cause mortality	1.16 (0.98–1.36)	Age at CRC diagnosis, sex, Prediagnostic BMI, smoking status, energy intake, tumor subsite, disease stage, and differentiation grade.
					Milk (Q4 vs. Q1, g/day)		1.21 (1.03–1.43)	
					Yogurt (Q4 vs. Q1, g/day)		1.08 (0.92–1.28)	
					Cheese (Q4 vs. Q1, g/day)		0.87 (0.74–1.04)	
					Total dairy (Q4 vs. Q1, g/day)	CRC-specific mortality	1.17 (0.96–1.43)	
					Milk (Q4 vs. Q1, g/day)		1.21 (0.99–1.48)	
					Yogurt (Q4 vs. Q1, g/day)		1.09 (0.88–1.34)	
					Cheese (Q4 vs. Q1, g/day)		0.93 (0.76–1.14)	
Dray 2003		Prediagnostic	64.4 [a] (10.2)	97/ 51	Meat (T3 vs. T1)	5-year survival rate	1.64 (0.75–3.58)	Age, sex, tumor stage, tumor location, and energy intake.
					Fish (T3 vs. T1)		1.24 (0.58–2.650	
					Eggs (T3 vs. T1)		1.10 (0.52–2.33)	
					Vegetables (T3 vs. T1)		1.09 (0.49–2.45)	
					Fruits (T3 vs. T1)		0.84 (0.37–1.88)	
					Rice and pasta (T3 vs. T1)		0.97 (0.44–2.15)	
					Bread (T3 vs. T1)		1.01 (0.43–2.33)	
					Sweet products (T3 vs. T1)		1.02 (0.48–2.16)	
					Tea and coffee (T3 vs. T1)		1.46 (0.64–3.22)	
					Dairy products (T3 vs. T1)		0.63 (0.30–1.33)	
					Carbohydrates (T3 vs. T1)		0.32 (0.14–0.76)	
					Proteins (T3 vs. T1)		0.27 (0.12–0.63)	

Study	Project, period (follow-up)	Diagnostic	Age (SD)	Sample size (male/ female)	Exposure	Outcome	RR/HR (95% CI)	Covariates
					Lipids (T3 vs. T1)		0.24 (0.09–0.59)	
					Fibre (T3 vs. T1)		1.87 (0.83–4.22)	
					Calcium (T3 vs. T1)		0.69 (0.28–1.70)	
					Iron (T3 vs. T1)		0.56 (0.21–1.46)	
					Copper (T3 vs. T1)		0.59 (0.26–1.34)	
					Zinc (T3 vs. T1)		0.92 (0.38–2.23)	
					Vitamin A (T3 vs. T1)		1.43 (0.71–2.88)	
					Beta-carotene (T3 vs. T1)		1.59 (0.74–3.43)	
					Vitamin B1 (T3 vs. T1)		0.75 (0.29–1.95)	
					Vitamin B2 (T3 vs. T1)		0.70 (0.31–1.59)	
					Vitamin B6 (T3 vs. T1)		1.02 (0.45–1.17)	
					Folic acid (T3 vs. T1)		1.67 (0.74–3.78)	
					Vitamin B12 (T3 vs. T1)		1.53 (0.73–3.21)	
					Vitamin C (T3 vs. T1)		1.11 (0.50–2.49)	
					Vitamin D (T3 vs. T1)		1.46 (0.73–2.93)	
					Vitamin E (T3 vs. T1)		1.43 (0.62–3.31)	
					Vitamin PP (T3 vs. T1)		1.15 (0.45–2.91)	
					Pantothenic acid (T3 vs. T1)		1.04 (0.44–2.44)	
					Biotine (T3 vs. T1)		1.12 (0.50–2.50)	
					Cholesterol (T3 vs. T1)		1.10 (0.47–2.61)	
					Saturated fatty acids (T3 vs. T1)		0.20 (0.08–0.49)	
					Monounsaturated fatty acids (T3 vs. T1)		0.35 (0.15–0.78)	
					Polyunsaturated fatty acids (T3 vs. T1)		0.52 (0.25–1.10)	
Fadelu 2018	CALGB 89803 (Alliance)	Postdiagnostic	60 [b]	466/ 360	Nut (≥2 servings/week vs. <1 serving/month)	All-cause mortality	0.43 (0.25–0.74)	Age, sex, depth of invasion through bowel wall, number of positive lymph nodes, baseline performance

Study	Project, period (follow-up)	Diagnostic	Age (SD)	Sample size (male/ female)	Exposure	Outcome	RR/HR (95% CI)	Covariates
								status, treatment group, BMI, physical activity, aspirin use, and glycemic load.
Fuchs 2014	CALGB 89803 (Alliance)	Postdiagnostic	58 [b]	569/ 442	Sugar-sweetened beverage (≥2 servings/day vs. <2 servings/month)	All-cause mortality	1.41 (0.79–2.50)	Age, sex, depth of invasion through bowel wall, number of positive lymph nodes, baseline performance status, treatment group, and the following time-varying covariates: total energy intake, BMI, physical activity level, Western dietary pattern, prudent dietary pattern, dietary glycemic load.
Fung 2014	NHS	Postdiagnostic	66.5 [b]	1,201	AHEI (Q5 vs. Q1, score)	All-cause mortality	0.71 (0.52–0.98)	Age, physical activity, BMI, weight change, cancer grade, chemotherapy, smoking status, energy intake, colon or rectal cancer, stage of disease, and date of CRC diagnosis.
					aMED (Q5 vs. Q1, score)		0.87 (0.63–1.21)	
					DASH (Q5 vs. Q1, score)		0.98 (0.71–1.35)	
					Prudent pattern (Q5 vs. Q1, score)		0.93 (0.65–1.34)	
					Western pattern (Q5 vs. Q1, score)		1.32 (0.89–1.97)	
					AHEI (Q5 vs. Q1, score)	CRC-specific mortality	0.72 (0.43–1.21)	
					aMED (Q5 vs. Q1, score)		0.84 (0.50–1.42)	
					DASH (Q5 vs. Q1, score)		0.87 (0.52–1.45)	
					Prudent pattern (Q5 vs. Q1, score)		0.67 (0.37–1.22)	
					Western pattern (Q5 vs. Q1, score)		1.66 (0.85–3.23)	
Guercio 2015	CALGB 89803 (Alliance)	Postdiagnostic	60.4 [b]	953/ 419	Coffee (≥4 cups/day vs. none)	All-cause mortality	0.66 (0.37–1.18)	Age, sex, depth of invasion through bowel wall, number of positive lymph nodes, baseline performance status, chemotherapy treatment group, smoking history, multivitamin, and the following time-varying covariates: total energy intake, alcohol consumption, BMI, physical activity level, Western dietary pattern, prudent dietary pattern, sugar-sweetened

Study	Project, period (follow-up)	Diagnostic	Age (SD)	Sample size (male/ female)	Exposure	Outcome	RR/HR (95% CI)	Covariates
								beverage intake and dietary glycemic load.
					Nonherbal tea (≥4 cups/day vs. none)		0.82 (0.40–1.67)	Age, sex, depth of invasion through bowel wall, number of positive lymph nodes, baseline performance status, chemotherapy treatment group, smoking history, multivitamin, total coffee intake and the following time-varying covariates: total energy intake, alcohol consumption, BMI, physical activity level, Western dietary pattern, prudent dietary pattern, sugar-sweetened beverage intake and dietary glycemic load.
Guercio 2018	CALGB 89803 (Alliance)	Postdiagnostic	64 [b]	574/ 444	Artificially sweetened beverage (≥1 serving/day vs. <2 servings/month)	All-cause mortality	0.52 (0.34–0.81)	Age, sex, depth of invasion through bowel wall, number of positive lymph nodes, baseline performance status, chemotherapy treatment group, consistent aspirin use, time-varying physical activity, total calorie intake, and BMI.
Guinter 2018	Cancer Prevention Study-II	Prediagnostic	73.6 [a] (7.0)	1,443/ 1,228	DASH (Q4 vs. Q1, score)	All-cause mortality	0.92 (0.78–1.08)	Age at diagnosis, year of diagnosis, sex, stage, total caloric intake, BMI, education, smoking status, and treatment.
					ACS (Q4 vs. Q1, score)		0.78 (0.65–0.95)	
					Prudent pattern (Q4 vs. Q1, score)		0.85 (0.73–1.00)	
					Western pattern (Q4 vs. Q1, score)		1.30 (1.03–1.64)	
					DASH (Q4 vs. Q1, score)	CRC-specific mortality	0.97 (0.74–1.28)	
					ACS (Q4 vs. Q1, score)		0.74 (0.54–1.03)	
					Prudent pattern (Q4 vs. Q1, score)		0.85 (0.64–1.13)	
					Western pattern (Q4 vs. Q1, score)		1.30 (0.88–1.91)	
		Postdiagnostic	70.6 [a] (6.1)	744/ 577	DASH (Q4 vs. Q1, score)	All-cause mortality	0.79 (0.62–0.99)	
					ACS (Q4 vs. Q1, score)		0.72 (0.56–0.93)	

Study	Project, period (follow-up)	Diagnostic	Age (SD)	Sample size (male/ female)	Exposure	Outcome	RR/HR (95% CI)	Covariates
					Prudent pattern (Q4 vs. Q1, score)		0.89 (0.81–0.98)	
					Western pattern (Q4 vs. Q1, score)		1.23 (0.91–1.65)	
					DASH (Q4 vs. Q1, score)	CRC-specific mortality	0.56 (0.35–0.89)	
					ACS (Q4 vs. Q1, score)		0.35 (0.17–0.73)	
					Prudent pattern (Q4 vs. Q1, score)		0.62 (0.37–1.06)	
					Western pattern (Q4 vs. Q1, score)		1.72 (0.95–3.12)	
Hu 2018	NHS and HPFS	Postdiagnostic	69 [a]	572/ 1,027	Coffee (≥4 cups/day vs. never)	All-cause mortality	0.70 (0.54–0.91)	Age at diagnosis, grade of differentiation, subsite, Postdiagnostic pack-years of smoking, alcohol consumption, BMI, physical activity, regular use of aspirin and NSAIDs and postmenopausal hormone use, folate, total fat, calcium, vitamin D, and sugar-sweetened beverages.
					Coffee (≥4 cups/day vs. never)	CRC-specific mortality	0.48 (0.28–0.83)	
Jayasekara 2018	Melbourne Collaborative Cohort Study	Prediagnostic	71 [b]	357/ 367	Alcohol (per 10g/day)	CRC-specific mortality	0.99 (0.91–1.08)	Age at diagnosis, year of diagnosis, sex, country of birth, American Joint Committee on Cancer stage, degree of differentiation, anatomic site, MSI status, BRAF status, KRAS status, cigarette smoking, exercising, walking, BMI, and waist circumference.
Keum 2017	NHS and HPFS	Prediagnostic	69 [a]	1,160	Glycemic score (1 SD)	CRC-specific mortality	1.02 (0.89–1.16)	Age at diagnosis, sex, year of diagnosis, cancer stage, questionnaire cycle, tumor grade, tumor location, post-diagnosis values of BMI, physical activity, smoking status, aspirin use, alcohol intake, and fiber intake.
					Glycemic load		1.10 (0.94–1.29)	
					Insulin score (1 SD)		1.19 (1.02–1.38)	
					Insulin load		1.23 (1.04–1.47)	
McCullough 2013	Cancer Prevention Study II	Prediagnostic	73 [a] (6.7)	1,282/ 1,033	Red and processed meat (Q4 vs. Q1, servings/week)	All-cause mortality	1.29 (1.05–1.59)	Age at diagnosis, sex, tumor stage at diagnosis, prediagnostic or postdiagnostic energy intake, weight change between 1992
					Red and processed meat (Q4 vs. Q1, servings/week)	CRC-specific mortality	1.09 (0.79–1.51)	

Study	Project, period (follow-up)	Diagnostic	Age (SD)	Sample size (male/ female)	Exposure	Outcome	RR/HR (95% CI)	Covariates
	Nutrition Cohort	Postdiagnostic			Red and processed meat (Q4 vs. Q1, servings/week)	All-cause mortality	0.94 (0.68–1.30)	prediagnostic and postdiagnostic questionnaires, and 1992 Prediagnostic meat intake.
					Red and processed meat (Q4 vs. Q1, servings/week)	CRC-specific mortality	1.10 (0.61–1.98)	
Meyerhardt 2007	CALGB 89803 (Alliance)	Postdiagnostic	60 [b]	566/ 443	Prudent diet (Q5 vs. Q1, score)	All-cause mortality	1.32 (0.86–2.04)	Age, sex, depth of invasion through bowel wall, number of positive lymph nodes (1–3 vs 4), presence of clinical perforation at time of surgery, presence of bowel obstruction at time of surgery, baseline performance status, treatment group, weight change between first and second questionnaire, time-varying BMI, time-varying physical activity level, and time-varying total calories.
					Western diet (Q5 vs. Q1, score)		2.32 (1.36–3.96)	
Meyerhardt 2012	CALGB 89803 (Alliance)	Postdiagnostic	60 [b]	569/ 442	Dietary glycemic load (Q5 vs. Q1)	All-cause mortality	1.74 (1.20–2.51)	Age, sex, depth of invasion through bowel wall, number of positive lymph nodes, baseline performance status, treatment group, time-varying BMI, time-varying physical activity level, time-varying cereal fiber and dietary pattern.
					Dietary glycemic index (Q5 vs. Q1)		1.23 (0.83–1.82)	
					Total fructose (Q5 vs. Q1)		1.11 (0.79–1.58)	
					Total carbohydrate (Q5 vs. Q1)		1.80 (1.25–2.60)	
Morales 2019	CALGB 89803 (Alliance)	Postdiagnostic	55 [b]	576/ 447	Dietary insulin load (Q5 vs. Q1)	All-cause mortality	2.30 (1.36–3.87)	Age, sex, depth of invasion through bowel wall, number of positive lymph nodes, tumor grade, baseline performance status, treatment group, BMI, physical activity, cereal fiber and alcohol intake. Mutually adjusted for glycemic load and index.
					Dietary insulin index (Q5 vs. Q1)		1.89 (1.22–2.91)	
Pelser 2014	NIH-AARP Diet and Health study	Prediagnostic	69.3 [a]	4,213 (colon cancer)	HEI-2005 (Q5 vs. Q1, score)	All-cause mortality	0.95 (0.78–1.16)	Age, lag time, sex, education, family history of colon cancer, cancer stage, first course of treatment (surgery, radiation, chemotherapy), and mutually adjusted for HEI-
					HEI-2005 (Q5 vs. Q1, score)	Colon mortality	0.99 (0.77–1.27)	
					Alcohol (heavy drinker vs. non-drinker)	All-cause mortality	0.92 (0.77–1.11)	

Study	Project, period (follow-up)	Diagnostic	Age (SD)	Sample size (male/ female)	Exposure	Outcome	RR/HR (95% CI)	Covariates
					Alcohol (heavy drinker vs. non-drinker)	Colon mortality	1.06 (0.84–1.33)	2005, BMI, physical activity, alcohol, and smoking history.
				1,514 (rectal cancer)	HEI-2005 (Q5 vs. Q1, score)	All-cause mortality	0.60 (0.42–0.86)	Age, lag time, sex, education, family history of colon cancer, cancer stage, first course of treatment (surgery, radiation, chemotherapy), and mutually adjusted for HEI-2005, BMI, physical activity, alcohol, and smoking history.
					HEI-2005 (Q5 vs. Q1, score)	Rectal mortality	0.64 (0.41–0.99)	
					Alcohol (heavy drinker vs. non-drinker)	All-cause mortality	1.03 (0.76–1.41)	
					Alcohol (heavy drinker vs. non-drinker)	Rectal mortality	0.97 (0.66–1.41)	
Phipps 2017	The Colon Cancer Family Registry (prospective)	Prediagnostic	-	2,582/ 2,384	Alcohol type (beer/hard cider vs. non-drinker/<1 drink/week)	All-cause mortality	0.93 (0.80–1.08)	Age at diagnosis, year of diagnosis, sex, smoking history, BMI, education, and study site.
					Alcohol type (wine/fortified wines vs. non-drinker/<1 drink/week)		0.81 (0.65–1.02)	
					Alcohol type (liquor vs. non-drinker/<1 drink/week)		0.89 (0.74–1.09)	
					Alcohol type (beer/hard cider vs. non-drinker/<1 drink/week)	CRC-specific mortality	1.01 (0.84–1.22)	
					Alcohol type (wine/fortified wines vs. non-drinker/<1 drink/week)		0.90 (0.68–1.20)	
					Alcohol type (liquor vs. non-drinker/<1 drink/week)		0.94 (0.73–1.21)	
Phipps 2016	N0147	Prediagnostic	58.0 [a] (11.2)	1,040/ 944	Lifetime alcohol consumption (ever-drinker vs. non-drinker)	All-cause mortality	0.85 (0.69–1.04)	Treatment, sex, BMI, smoke, physical activity, performance score, and race.
Ratjen 2017	PopGen CRC survivor cohort	Postdiagnostic	69 [b]	788/ 616	MMED (Q4 vs. Q1, score)	All-cause mortality	0.48 (0.32–0.74)	Age at diet assessment, sex, BMI, physical activity, survival time from CRC diagnosis until diet assessment, tumor location, occurrence of metastases, occurrence of other cancer, chemotherapy, smoking status, total energy intake, time*age, time*BMI, and time*metastases.
					HNFI (Q4 vs. Q1, score)		0.63 (0.39–1.04)	
Ratjen 2019	PopGen CRC survivor cohort	Postdiagnostic	62 [b]	788/ 616	DII (Q4 vs. Q1, score)	All-cause mortality	1.36 (0.88–2.09)	Age at diet assessment, sex, BMI, physical activity, survival time from

Study	Project, period (follow-up)	Diagnostic	Age (SD)	Sample size (male/ female)	Exposure	Outcome	RR/HR (95% CI)	Covariates
								CRC diagnosis until diet assessment, tumor location, occurrence of metastases, occurrence of other cancer, chemotherapy, smoking status, total energy intake, time*age, time*BMI, and time*metastases.
Romaguera 2015	EPIC	Prediagnostic	64.6 [a] (8.0)	1,794/ 1,498	WCRF/AICR score (Q4 vs. Q1)	CRC-specific mortality	0.70 (0.56–0.89)	Age at CRC diagnosis, year of CRC diagnosis, tumor stage, tumor grade, tumor size, sex, educational level, and smoking status.
					WCRF/AICR score (Q4 vs. Q1)	All-cause mortality	0.79 (0.65–0.98)	
Sauvaget 2003	Life Span Study (LSS)	Prediagnostic	56 [a]	14,873/ 23,667	Green-yellow vegetables (daily or almost daily vs. once per week or less)	All-cause mortality	0.92 (0.84–1.01)	Age, sex, radiation dose, city, BMI, smoking status, alcohol habits, and education level.
					Green-yellow vegetables (daily or almost daily vs. once per week or less)	CRC-specific mortality	1.10 (0.82–1.47)	
					Fruits (daily or almost daily vs. once per week or less)	All-cause mortality	0.88 (0.80–0.96)	
					Fruits (daily or almost daily vs. once per week or less)	CRC-specific mortality	0.97 (0.73–1.29)	
Sharma 2018	NFCCR (Newfoundland Familial Colorectal Cancer Registry)	Prediagnostic	62.5 [a] (9.1)	434/ 268	Processed meat pattern	All-cause mortality	1.53 (0.85–2.27)	Age, sex, marital status, tumor location, screening history, intake of alcohol and energy, stage of cancer, radiation and chemo therapy status, and MSI status wherever applicable.
					Prudent vegetable pattern		1.03 (0.61–1.75)	
					High-sugar pattern		1.27 (0.72–2.23)	
					DII (Q4 vs. Q1, score)		0.78 (0.47–1.25)	
					aMED (Q4 vs. Q1, score)		0.62 (0.39–0.97) [c]	
					Recommended Food Score (Q4 vs. Q1, score)		0.65 (0.60–1.67) [c]	
Skeie 2014	HELGA cohort	Prediagnostic	-	529 (male)	Total whole grain (T3 vs. T1, g/day)	CRC-specific mortality	1.00 (0.67–1.48)	Age at diagnosis, metastasis, smoking, and intake of folate, margarine, and energy, and mutually adjusted for the other grains.
					Wheat (T3 vs. T1, g/day)		0.97 (0.64–1.49)	
					Rye (T3 vs. T1, g/day)		0.90 (0.60–1.36)	
					Oats (T3 vs. T1, g/day)		1.11 (0.72–1.70)	

Study	Project, period (follow-up)	Diagnostic	Age (SD)	Sample size (male/ female)	Exposure	Outcome	RR/HR (95% CI)	Covariates
					Whole grain products (T3 vs. T1, g/day)		1.06 (0.71–1.56)	
				590 (female)	Total whole grain (T3 vs. T1, g/day)		0.91 (0.60–1.39)	
					Wheat (T3 vs. T1, g/day)		1.35 (0.72–2.53)	
					Rye (T3 vs. T1, g/day)		0.93 (0.60–1.46)	
					Oats (T3 vs. T1, g/day)		0.83 (0.55–1.26)	
					Whole grain products (T3 vs. T1, g/day)		1.10 (0.74–1.64)	
Song 2017	NHS and HPFS	Postdiagnostic	68.7 [a]	1,659	Marine w-3 polyunsaturated fatty acid (≥0.30g/day vs. <0.1g/day)	All-cause mortality	0.95 (0.73–1.25)	Age at diagnosis, sex, cancer stage, Prediagnostic intake of marine w-3 polyunsaturated fatty acids, grade of differentiation, subsite, pack-years of smoking, alcohol consumption, BMI, physical activity, regular use of aspirin and NSAIDs, postmenopausal hormone use, and intake of folate and vitamin D.
					Marine w-3 polyunsaturated fatty acid (≥0.30g/day vs. <0.1g/day)	CRC-specific mortality	0.59 (0.35–1.01)	
Song 2018	NHS and HPFS	Postdiagnostic	68.6 [a] (8.9)	612/ 963	Fiber (Q4 vs. Q1, g/day)	All-cause mortality	0.64 (0.51–0.80)	Age at diagnosis, sex, cancer stage, year of diagnosis, tumor grade of differentiation, subsite, Prediagnostic fiber intake, Postdiagnostic alcohol consumption, pack-years of smoking, BMI, physical activity, regular use of aspirin, glycemic load, and consumption of total fat, folate, calcium, vitamin D, and fiber. *Mutually adjusted for fiber intake from other sources.
					Fiber (Q4 vs. Q1, g/day)	CRC-specific mortality	0.54 (0.35–0.85)	
					Whole grains (Q4 vs. Q1, g/day)*	All-cause mortality	0.81 (0.65–1.01)	
					Whole grains (Q4 vs. Q1, g/day)*	CRC-specific mortality	0.57 (0.35–0.92)	
Song 2019a	NHS and HPFS	Postdiagnostic	68.0 [a] (9.3)	617/ 925	Fat (Q4 vs. Q1)*	All-cause mortality	0.87 (0.72–1.07)	Age at diagnosis, cancer stage, Postdiagnostic intake of total calorie, year of diagnosis, subsite, Prediagnostic intake of the nutrient
					Saturated fat (Q4 vs. Q1)		0.93 (0.74–1.18)	
					Polyunsaturated fat (Q4 vs. Q1)		0.81 (0.66–0.98)	

Study	Project, period (follow-up)	Diagnostic	Age (SD)	Sample size (male/ female)	Exposure	Outcome	RR/HR (95% CI)	Covariates
					Monounsaturated fat (Q4 vs. Q1)		0.89 (0.69–1.14)	under analysis, Postdiagnostic alcohol consumption, pack-years of smoking, BMI, physical activity, regular use of aspirin, and total fibre intake. *Additionally adjusted for Postdiagnostic total protein inke †Additionally adjusted for Postdiagnostic total fat inke
					Protein (Q4 vs. Q1)†		1.24 (1.03–1.49)	
					Carbohydrate (Q4 vs. Q1)*		1.21 (0.97–1.52)	
					Fat (Q4 vs. Q1)*	CRC-specific mortality	0.68 (0.44–1.06)	
					Saturated fat (Q4 vs. Q1)		1.30 (0.77–2.19)	
					Polyunsaturated fat (Q4 vs. Q1)		0.72 (0.48–1.09)	
					Monounsaturated fat (Q4 vs. Q1)		0.87 (0.50–1.53)	
					Protein (Q4 vs. Q1)†		1.01 (0.70–1.46)	
					Carbohydrate (Q4 vs. Q1)*		1.91 (1.17–3.12)	
Song 2019b	N0147 (Alliance)	Prediagnostic	58.01 [a]	897/ 838	Marine w-3 polyunsaturated fatty acid (Q4 vs. Q1, g/day)	All-cause mortality	0.93 (0.70–1.23)	Age, sex, study arm, tumor subsite, number of affected nodes, T stage, ECOG performance score, aspirin use, MMR status, smoking status, alcohol consumption, BMI, frequency of vigorous physical activity, multivitamin use, and total intake of vitamin D, calcium, fiber, and processed red meat.
Sun 2019		Prediagnostic	58.46 [a]	188/ 124	Whole grain (Q4 vs. Q1, g/day)	CRC-specific mortality	0.56 (0.35–0.89)	Age, sex, BMI, smoking, drinking, and energy intake.
					Fruit (Q4 vs. Q1, g/day)		0.62 (0.40–0.97)	
					Red meat (Q4 vs. Q1, g/day)		1.68 (1.08–2.61)	
					Coffee habit (yes vs. no)		0.46 (0.24–0.87)	
					Grilled food (7 times/week vs. 1 time/month)		1.78 (1.05–3.02)	
					Diet quality score (T3 vs. T1)		0.63 (0.32–1.21) [c]	Age, sex, BMI, smoking, drinking, energy intake, UICC stage, tumor size, chemotherapy, postoperative adjuvant radiotherapy, CA19–9 and CEA.
	CALGB 89803 (Alliance)	Postdiagnostic	60.3 [b]	569/ 442	Marine w-3 polyunsaturated fatty acid (Q4 vs. Q1, g/day)	All-cause mortality	0.81 (0.58–1.15)	Age, sex, energy intake, T stage, number of positive lymph nodes,

Study	Project, period (follow-up)	Diagnostic	Age (SD)	Sample size (male/ female)	Exposure	Outcome	RR/HR (95% CI)	Covariates
Van Blarigan 2018a					Dark fish (≥1/week vs. never, 3–5 oz.)		0.68 (0.48–0.96)	baseline performance status, treatment arm, BMI, physical activity, smoking, and aspirin use.
					Canned tuna fish (≥1/week vs. never, 3–4 oz.)		1.23 (0.82–1.84)	
					Other fish (≥1/week vs. never, 3–5 oz.)		1.03 (0.71–1.48)	
					Breaded fish cakes, pieces, or sticks (≥1/week vs. never, 1 serving, store bought)		1.10 (0.74–1.64)	
					Shrimp, lobster, scallops, clams as a main dish (≥1/week vs. never, 1 serving)		1.04 (0.73–1.50)	
Van Blarigan 2018b	CALGB 89803 (Alliance)	Postdiagnostic	59.6 [a] (11.2)	562/ 430	ACS guidelines score (5–6 vs. 0–1)	All-cause mortality	0.58 (0.34–0.99)	Age, sex, total energy intake, T stage, number of positive lymph nodes, baseline performance status, treatment arm, smoking status, and aspirin use.
					ACS guidelines score including alcohol consumption (6–8 vs. 0–2)		0.49 (0.32–0.76)	
Walter 2016	DACHS	Prediagnostic	69 [b]	1,855/ 1,266	Lifetime alcohol (heavy drinker vs. light drinker)	All-cause mortality	1.37 (1.06–1.78)	Age, sex, stage, smoking status, use of statins, use of NSAIDs, use of beta-blockers, diabetes mellitus, history of heart failure, myocardial infarction, angina pectoris, or stroke, history of Ca, age*log(time), and Ca*log(time). *Mutually adjusted for other beverage intake.
					Recent alcohol (heavy drinker vs. light drinker)		1.17 (0.91–1.51)	
					Lifetime alcohol (heavy drinker vs. light drinker)	CRC-specific mortality	1.18 (0.85–1.62)	
					Recent alcohol (heavy drinker vs. light drinker)		1.03 (0.76–1.39)	
Ward 2016	EPIC	Prediagnostic	-	1,603 /2,186	Fiber (Q4 vs. Q1, g/day)	All-cause mortality	0.84 (0.66–1.06)	Age at diagnosis, sex, BMI, smoking status, tumor grade, tumor stage, year of tumor diagnosis, energy intake, Ca intake, folate intake, alcohol intake, and education.
					Red and processed meat (Q4 vs. Q1, g/day)		1.00 (0.83–1.20)	
					Red meat (Q4 vs. Q1, g/day)		0.95 (0.78–1.14)	
					Processed meat (Q4 vs. Q1, g/day)		1.17 (0.97–1.42)	
					Poultry (Q4 vs. Q1, g/day)		0.87 (0.73–1.03)	
					Fiber (Q4 vs. Q1, g/day)	CRC-specific mortality	0.90 (0.69–1.17)	
					Red and processed meat (Q4 vs. Q1, g/day)		1.00 (0.81–1.23)	

Study	Project, period (follow-up)	Diagnostic	Age (SD)	Sample size (male/female)	Exposure	Outcome	RR/HR (95% CI)	Covariates
					Red meat (Q4 vs. Q1, g/day)		0.93 (0.75–1.15)	
					Processed meat (Q4 vs. Q1, g/day)		1.12 (0.90–1.39)	
					Poultry (Q4 vs. Q1, g/day)		0.91 (0.75–1.10)	
Yang 2014	Cancer Prevention Study-II Nutrition Cohort	Prediagnostic	73 [a]	1,274/1,010	Calcium (Q4 vs. Q1, mg/day)	All-cause mortality	0.86 (0.71–1.04)	Age at diagnosis, sex, tumor stage, post-diagnosis total energy intake, and total folate intake.
					Vitamin D (Q4 vs. Q1, IU/day)		0.97 (0.80–1.17)	
					Total dairy (Q4 vs. Q1, serving/week)		0.88 (0.72–1.09)	
					Milk (Q4 vs. Q1, serving/week)		0.95 (0.79–1.15)	
					Calcium (Q4 vs. Q1, mg/day)	CRC-specific mortality	0.86 (0.64–1.16)	
					Vitamin D (Q4 vs. Q1, IU/day)		0.96 (0.72–1.28)	
					Total dairy (Q4 vs. Q1, serving/week)		0.89 (0.65–1.22)	
					Milk (Q4 vs. Q1, serving/week)		0.98 (0.73–1.32)	
		Postdiagnostic			Calcium (Q4 vs. Q1, mg/day)	All-cause mortality	0.86 (0.65–1.14)	
					Vitamin D (Q4 vs. Q1, IU/day)		0.90 (0.67–1.21)	
					Total dairy (Q4 vs. Q1, serving/week)		0.75 (0.56–1.01)	
					Milk (Q4 vs. Q1, serving/week)		0.72 (0.55–0.94)	
					Calcium (Q4 vs. Q1, mg/day)	CRC-specific mortality	1.00 (0.61–1.63)	
					Vitamin D (Q4 vs. Q1, IU/day)		1.28 (0.77–2.10)	
					Total dairy (Q4 vs. Q1, serving/week)		0.73 (0.44–1.23)	
					Milk (Q4 vs. Q1, serving/week)		0.93 (0.59–1.49)	
Yang 2016	Cancer Prevention Study-II Nutrition Cohort	Postdiagnostic	68 [a]	1,660	Calcium (Q4 vs. Q1, mg/day)	All-cause mortality	0.80 (0.59–1.09)	Age at diagnosis, cancer stage, sex, tumor grade of differentiation, tumor subsite, year of diagnosis, Prediagnostic intake of total calcium, Postdiagnostic BMI, Postdiagnostic physical activity, Postdiagnostic regular use of aspirin, Postdiagnostic smoking, Postdiagnostic alcohol consumption, Postdiagnostic intake
					Calcium (Q4 vs. Q1, mg/day)	CRC-specific mortality	0.56 (0.32–0.96)	

Study	Project, period (follow-up)	Diagnostic	Age (SD)	Sample size (male/ female)	Exposure	Outcome	RR/HR (95% CI)	Covariates
								of total fat, folate, and vitamin D, and multivitamin and other supplement use.
Yang 2017	Cancer Prevention Study-II Nutrition Cohort	Prediagnostic	-	1,370/ 1,088	Alcohol (current ≥3 drinks/day vs. never)	All-cause mortality	1.11 (0.83–1.47)	Age at diagnosis, sex, tumor stage at diagnosis, smoking status, BMI, physical activity, education, and preexisting diseases in 1982/1992 (chronic obstructive pulmonary disease, liver disease, or kidney disease).
					Alcohol (current ≥3 drinks/day vs. never)	CRC-specific mortality	1.23 (0.77–1.98)	
		Postdiagnostic			Alcohol (current ≥2 drinks/day vs. never)	All-cause mortality	0.92 (0.66–1.28)	
					Alcohol (current ≥2 drinks/day vs. never)	CRC-specific mortality	1.44 (0.80–2.60)	
Yuan 2017	NHS and HPFS	Postdiagnostic	67.2 [a]	692/ 1,314	Dietary insulin load (Q5 vs. Q1)	All-cause mortality	1.33 (1.03–1.72)	Age at diagnosis, sex, race, smoking status, BMI, physical activity, alcohol intake, cancer stage, grade of tumor differentiation, location of primary tumor, and year of diagnosis.
					Dietary insulin load (Q5 vs. Q1)	CRC-specific mortality	1.82 (1.20–2.75)	
					Dietary insulin index (Q5 vs. Q1)	All-cause mortality	1.32 (1.02–1.71)	
					Dietary insulin index (Q5 vs. Q1)	CRC-specific mortality	1.66 (1.10–2.50)	
Zamora-Ros 2015	Bellvitge colorectal cancer study	Prediagnostic	-	437/ 291	Total flavonoids (T3 vs. T1, mg/day)	All-cause mortality	0.97 (0.60–1.56)	Age, sex, total energy intake, and CRC stage.
					Flavanols (T3 vs. T1, mg/day)		0.99 (0.63–1.58)	
					Flavan-3-ol monomers (T3 vs. T1, mg/day)		0.93 (0.59–1.46)	
					Proanthocyanidins (T3 vs. T1, mg/day)		1.08 (0.68–1.71)	
					Flavonols (T3 vs. T1, mg/day)		0.90 (0.58–1.39)	
					Flavanones (T3 vs. T1, mg/day)		0.92 (0.60–1.42)	
					Anthocyanidins (T3 vs. T1, mg/day)		0.91 (0.58–1.44)	
					Flavones (T3 vs. T1, mg/day)		0.87 (0.56–1.36)	
					Isoflavones (T3 vs. T1, mg/day)		0.97 (0.62–1.53)	
					Lignans (T3 vs. T1, mg/day)		0.83 (0.50–1.37)	
					Total flavonoids (T3 vs. T1, mg/day)	CRC-specific mortality	0.87 (0.47–1.62)	
					Flavanols (T3 vs. T1, mg/day)		1.34 (0.73–2.45)	

Study	Project, period (follow-up)	Diagnostic	Age (SD)	Sample size (male/ female)	Exposure	Outcome	RR/HR (95% CI)	Covariates
					Flavan-3-ol monomers (T3 vs. T1, mg/day)		0.91 (0.49–1.67)	
					Proanthocyanidins (T3 vs. T1, mg/day)		1.30 (0.71–2.39)	
					Flavonols (T3 vs. T1, mg/day)		1.18 (0.65–2.13)	
					Flavanones (T3 vs. T1, mg/day)		0.80 (0.46–1.39)	
					Anthocyanidins (T3 vs. T1, mg/day)		0.87 (0.48–1.57)	
					Flavones (T3 vs. T1, mg/day)		0.97 (0.54–1.73)	
					Isoflavones (T3 vs. T1, mg/day)		0.60 (0.33–1.09)	
					Lignans (T3 vs. T1, mg/day)		0.68 (0.36–1.26)	
Zell 2007	Irvine Gene-Environment Study of Familial CRC	Prediagnostic	-	141 (familial CRC)	Beer (regular vs. infrequent)	CRC-specific mortality	1.07 (0.50–2.29)	Age, sex, treatment with surgery, radiation, and chemotherapy.
					Liquor (regular vs. infrequent)		1.13 (0.56–2.29)	
					Wine (regular vs. infrequent)		0.50 (0.25–0.99)	
				358 (sporadic CRC)	Beer (regular vs. infrequent)		1.01 (0.65–1.57)	
					Liquor (regular vs. infrequent)		0.79 (0.51–1.24)	
					Wine (regular vs. infrequent)		0.89 (0.59–1.33)	
Zell 2010	California Teachers Study	Prediagnostic	66.2 [b]	-	Meat (9.15–29.52 medium-sized servings/week vs. 0–5.39 medium sized servings/week)	CRC-specific mortality	0.97 (0.85–1.10)	Age at baseline questionnaire (years) and stage, family history of CRC in a first-degree relative, site (colon or rectum), treatment with surgery, and total daily energy intake (kcal/day).
Zheng 2020	WHI	Postdiagnostic	-	463	Energy-adjusted DII (T3 vs. T1)	All-cause mortality	1.39 (0.89–2.17) [c]	Age at baseline, race/ethnicity, smoking status at baseline, income levels, cancer stage, education, years from cancer diagnosis to FFQ, baseline physical activity in MET-h/week, total energy intake per day, BMI at baseline, cancer differentiation grading, supplement intake, and time-dependent status
					Energy-adjusted DII (T3 vs. T1)	CRC-specific mortality	1.33 (0.64–2.78) [c]	

Study	Project, period (follow-up)	Diagnostic	Age (SD)	Sample size (male/ female)	Exposure	Outcome	RR/HR (95% CI)	Covariates
								before and after post-diagnosis FFQ.
Zhu 2013	NFCCR	Prediagnostic	60.4 [a]	318/ 211	Processed meat pattern (Q4 vs. Q1)	CRC-specific mortality	1.53 (0.85–2.74)	Age at diagnosis, sex, total energy intake, stage at diagnosis, marital status, family history, reported screening procedure, reported chemoradiotherapy, and microsatellite instability status, where appropriate.
					Prudent vegetable pattern (Q4 vs. Q1)		1.03 (0.61–1.75)	
					High-sugar pattern (Q4 vs. Q1)		1.27 (0.72–2.25)	
					Processed meat pattern (Q4 vs. Q1)	Colon mortality	2.13 (1.03–4.43)	
					Prudent vegetable pattern (Q4 vs. Q1)		0.96 (0.47–1.96)	
					High-sugar pattern (Q4 vs. Q1)		1.16 (0.54–2.47)	
					Processed meat pattern (Q4 vs. Q1)	Rectal mortality	1.17 (0.41–3.36)	
					Prudent vegetable pattern (Q4 vs. Q1)		1.00 (0.42–2.40)	
					High-sugar pattern (Q4 vs. Q1)		1.68 (0.55–5.08)	

CRC, colorectal cancer; SD, standard deviation; Q, quartile or quintile; T, tertile; BMI, body mass index; aHEI, alternate Healthy Eating Index; aMED, alternate Mediterranean Diet score; eDII, energy-adjusted Dietary Inflammatory Index; mMED, Modified Mediterranean Diet score; HNFI, Healthy Nordic Food Index; DASH, Dietary Approaches to Stop Hypertension score; ACS, American Cancer Society nutrition guidelines; NSAIDs, Non-steroidal anti-inflammatory drugs; HEI, Healthy Eating Index; MSI, microsatellite instability; WCRF/AICR, World Cancer Research Fund/American Institute of Cancer Research; ECOG, Eastern Cooperative Oncology Group; MMR, mismatch repair; UICC, International Union Against Cancer; CA19-9, preoperative carbohydrate antigen; CEA, carcinoembryonic antigen; DII, Dietary Inflammatory Index; MET, metabolic equivalent; FFQ, food frequency questionnaire. [a] Age is presented as the mean value [b] Age is presented as the median value [c] The 95% CI was recalculated from different levels of significant α using the following formula: $(1\quad \alpha)\%\ CI\ for\ HR = exp[\hat{\beta}];\ exp[\hat{\beta} \pm z_{1-\alpha/2} * \sqrt{Var(\hat{\beta})}$

Table S4. Newcastle-Ottawa quality assessment scale of the cohort studies included in the systematic review and meta-analysis.

	Selection				Comparability		Outcome			
**Study**	**Representativeness of the exposed cohort**	**Selection of the nonexposed cohort**	**Ascertainment of exposure**	**Outcome of interest not present at start**	**Age and sex**	**Additional factors**	**Assessment of outcome**	**Follow-up length**	**Adequacy of follow-up of cohorts**	**Total**
Brown 2018	1	1	0	1	1	1	1	1	1	8
Carr 2016	1	1	0	1	1	1	1	1	1	8
Carr 2019	1	1	0	1	1	1	1	1	1	8
Dik 2014	1	1	0	1	1	1	1	1	1	8
Dray 2003	0	1	1	1	1	1	1	1	1	8
Fadelu 2018	1	1	0	1	1	1	1	1	1	8
Fuchs 2014	1	1	0	1	1	1	1	1	1	8
Fung 2014	1	1	0	1	0	1	1	1	1	7
Guercio 2015	1	1	0	1	1	1	1	1	1	8
Guercio 2018	1	1	0	1	1	1	1	1	1	8
Guinter 2018	1	1	0	1	1	1	1	1	1	8
Hu 2018	1	1	0	1	0	1	1	1	1	7
Jayasekara 2018	1	1	0	1	1	1	1	1	1	8
Keum 2017	1	1	0	1	1	1	1	1	1	8
McCullough 2013	1	1	0	1	1	1	1	1	1	8
Meyerhardt 2007	1	1	0	1	1	1	1	1	1	8
Meyerhardt 2012	1	1	0	1	1	1	1	1	1	8
Morales 2019	1	1	0	1	1	1	1	1	1	8
Pelser 2014	1	1	0	1	1	1	1	1	1	8
Phipps 2016	1	1	0	1	1	1	1	1	1	8
Phipps 2017	1	1	0	1	1	1	1	0	1	7
Ratjen 2017	1	1	0	1	1	1	1	1	1	8
Ratjen 2019	1	1	0	1	1	1	1	1	1	8
Romaguera 2015	1	1	0	1	1	1	1	1	1	8
Sauvaget 2003	1	1	0	1	1	1	1	1	1	8
Sharma 2018	1	1	0	1	1	1	1	1	1	8
Skeie 2014	1	1	0	1	1	1	1	1	1	8
Song 2017	1	1	0	1	1	1	1	1	1	8

Study	Selection				Comparability		Outcome			Total
	Representativeness of the exposed cohort	Selection of the nonexposed cohort	Ascertainment of exposure	Outcome of interest not present at start	Age and sex	Additional factors	Assessment of outcome	Follow-up length	Adequacy of follow-up of cohorts	
Song 2018	1	1	0	1	1	1	1	1	1	8
Song 2019a	1	1	0	1	1	1	1	1	1	8
Song 2019b	1	1	0	1	1	1	1	1	1	8
Sun 2019	1	1	0	1	1	1	0	1	1	7
Van Blarigan 2018a	1	1	0	1	1	1	1	1	1	8
Van Blarigan 2018b	1	1	0	1	1	1	1	1	1	8
Walter 2016	1	1	0	1	1	1	1	1	1	8
Ward 2016	1	1	0	1	1	1	1	1	1	8
Yang 2014	1	1	0	1	1	1	1	1	1	8
Yang 2016	1	1	0	1	1	1	1	1	1	8
Yang 2017	1	1	0	1	1	1	1	1	1	8
Yuan 2017	1	1	0	1	1	1	1	1	1	8
Zamora-Ros 2015	1	1	0	1	1	1	1	1	1	8
Zell 2007	1	1	0	1	1	1	1	1	1	8
Zell 2010	1	1	0	1	1	1	1	1	1	8
Zheng 2020	1	1	0	1	1	1	1	1	1	8
Zhu 2013	1	1	0	1	1	1	1	1	1	8

Table S5. Assessment of attribution bias in the individual studies.

Study	Attrition bias	Judgement
Brown 2018	Unknown	Adjustment for censoring data: "Patients who died without known disease recurrence were censored at the last documented physician evaluation."
Carr 2016	Low	Adjustment for censoring data: "Patients who did not have an endpoint of interest were censored at the last contact they were known to be alive or free of recurrence" and "In cases in which the patient died before follow-up or did not complete the follow-up questionnaire, information on cancer recurrence before death was collected from the last at tending physicians."<br>Completeness of follow-up is reported as a strength in this study.

Study	Attrition bias	Judgement
Carr 2019	Unknown	Adjustment for censoring data: "Patients alive at the most recent follow-up or data linkage were censored on that date in the survival analysis."
Dik 2014	Unknown	Adjustment for censoring data: "The date of censoring was defined as the last date at which follow-up data were judged to be complete, the last date of contact, or date of death."
Dray 2003	Low	Censoring was reported: "None was lost to follow-up."
Fadelu 2018	Low	Censoring was reported: 39/826 censored for cancer recurrence.
Fuchs 2014	Low	Censoring was reported: 43/1,011 censored for cancer recurrence.
Fung 2014	Unknown	Adjustment for censoring data: "Those who died without known tumor recurrence were included in the analysis of overall mortality but not the analysis of colorectal cancer mortality."
Guercio 2015	Low	Censoring was reported: 36/953 censored for cancer recurrence.
Guercio 2018	Low	Censoring was reported: 44/1,018 censored for cancer recurrence.
Guinter 2018	Low	Adjustment for inverse causality: "In the postdiagnosis models, participants who completed their FFQ within 12 months of CRC diagnosis were excluded to account for the potential influence of adverse treatment effects on diet, and in another model, the first 2 years of follow-up after completion of the postdiagnosis FFQ were excluded."
Hu 2018	Low	Response rate was reported: "The response rates for the NHS and HPFS exceeded 90%."
Jayasekara 2018	Low	Adjustment for censoring data: "For CRC-specific survival, individuals were censored at death when the cause of death was anything other than CRC, but were excluded when no information was available on cause of death."<br>Censoring was reported: 23/339 censored for death.
Keum 2017	Low	Adjustment for censoring data: "In the primary analysis, CRC mortality was the main endpoint, and deaths from other causes were censored."<br>High response rate was a strength of the study: "Our study has several strengths, including prospective design, high follow-up rates and the ability to adjust for a variety of confounders."
McCullough 2013	Low	Adjustment for reverse causality: "Sensitivity analyses excluded the first 2 years of follow-up after diagnosis and excluded patients with a history of heart attack, stroke, or lung disease."
Meyerhardt 2007	Low	Censoring was reported: 28/1,009 censored for cancer recurrence.
Meyerhardt 2012	Low	Censoring was reported: 43/1,011 censored for cancer recurrence.

Study	Attrition bias	Judgement
Morales 2019	Low	Adjustment for censoring data: "Recurrence-free survival (RFS) was defined as time from completion of Q1 to colon cancer recurrence or occurrence of a new primary colon cancer, or death with evidence of recur rence; patients who died without evidence of recurrence were censored at last date of evaluation."
Pelser 2014	Low	Adjustment for censoring data: "Exploratory analyses revealed interactions between lag time and the composite lifestyle score, but limiting follow-up time to 5 years after diagnosis eliminated this interaction."
Phipps 2016	Low	Adjustment for censoring data: "Patients were censored at 5 years postrandomization in analyses of DFS and TTR; patients were censored at 8 years postrandomization for OS analyses." and "Censoring events were reaching 5 years follow-up after diagnosis, or at study end."
Phipps 2017	Low	Adjustment for censoring data: "Our timescale was defined as days since the CRC diagnosis, with left censoring to account for the time lag between diagnosis and C-CFR enrollment." and "In analyses of CRC-specific survival, we censored participants who died from causes other than CRC."
Ratjen 2017	High	Sufficiently high proportion of excluding subjects: 1,404 in total of 2,733 subjects remained after third follow-up.
Ratjen 2019	High	Response rate or censoring adjustment was not reported.
Romaguera 2015	Unknown	Adjustment for censoring data: "Age was used as the primary time variable, with entry time defined as the subject's age at CRC diagnosis and exit time as age censoring or death."
Sauvaget 2003	Unknown	Response rate of 72.5% was reported.
Sharma 2018	Unknown	Adjustment for censoring data: "Individuals who were lost to follow up, still alive or who did not have a recurrence or metastasis by the end of the follow-up period were censored at the time of the last contact."
Skeie 2014	Unknown	Adjustment for censoring data: "Censoring for total deaths was done on the same date."
Song 2017	Low	Follow-up rate was reported: "The follow-up rates have been 95.4% in the NHS and 95.9% in the HPFS for each of the questionnaires through 2010."
Song 2018	Low	Follow-up rate was reported: "The follow-up rates have been 95.4% in the NHS and 95.9% in the HPFS for each of the questionnaires through 2010."
Song 2019a	Low	Follow-up rate was reported: "The follow-up rates have been 95.4% in the NHS and 95.9% in the HPFS for each of the questionnaires through 2010."
Song 2019b	Low	Follow-up rate was reported: "The follow-up rate in the two cohorts has been higher than 90% for each questionnaire cycle."
Sun 2019	Low	40/352 (11%) lost to follow-up was reported.

Study	Attrition bias	Judgement
Van Blarigan 2018a	Low	Completement of follow-up was reported: "Patients in CALGB 89803 were followed for a minimum of 7 years after treatment; median follow-up was 7 years and nearly 100% complete."
Van Blarigan 2018b	Low	Adjustment for censoring data: "Recurrence-free survival was defined as time to tumor recurrence or new primary colon tumor; patients who died without recurrence were censored."<br>High follow-up rate was reported: "Follow-up included nearly 100% of enrollees."
Walter 2016	High	Response rate or censoring adjustment was not reported.
Ward 2016	Low	High follow-up rate was reported: "Loss to follow-up across all countries was low (<2%)."
Yang 2014	Low	Adjustment for inverse causality: "Because postdiagnosis diet and supplement use may be influenced by serious illness preceding death (reverse causation), we conducted a sensitivity analysis excluding deaths within the first 2 years of follow-up after completion of the postdiagnosis questionnaire."
Yang 2016	Low	Sensitivity analysis of excluding the first 2 years of follow-up was conducted to adjust for reverse causality.
Yang 2017	Low	Adjustment for inverse causality: "In sensitivity analyses, the first 2 years of follow-up for prediagnosis and postdiagnosis models were excluded."
Yuan 2017	Low	High follow-up rate was reported: "The current study has several strengths, including the prospective design, large sample size, long follow-up period, high follow-up rate, and detailed information on other prognostic factors."
Zamora-Ros 2015	Unknown	Follow-up rate was reported: "Inherent to prospective studies, we had some lost of follow-up, but more than 75% of the patients had at least 5 years of information."
Zell 2007	Unknown	Adjustment for censoring data: "The last date of follow-up was either the date of death or the last date the patient was contacted."
Zell 2010	Low	Adjustment for censoring data: "Patients with death from causes other than CRC were censored, as were patients who survived to the end of the follow-up period."
Zheng 2020	Unknown	Adjustment for censoring data: "Each participant accumulated person-years of follow-up time from diagnosis of primary colorectal cancer until death, loss to follow-up, the last NDI search date for the participant, or the end of follow-up (October 2014), whichever occurred first."
Zhu 2013	Unknown	Adjustment for censoring data: "Patients who did not have an event by the end of the follow-up were censored at the date of last contact."

© 2020 by the authors. Licensee MDPI, Basel, Switzerland. This article is an open access article distributed under the terms and conditions of the Creative Commons Attribution (CC BY) license (http://creativecommons.org/licenses/by/4.0/).
